# Chemical Conjugation in Drug Delivery Systems

**DOI:** 10.3389/fchem.2022.889083

**Published:** 2022-05-26

**Authors:** Alexis Eras, Danna Castillo, Margarita Suárez, Nelson Santiago Vispo, Fernando Albericio, Hortensia Rodriguez

**Affiliations:** ^1^ School of Chemical Sciences and Engineering, Yachay Tech University, Urcuquí, Ecuador; ^2^ Laboratorio de Síntesis Orgánica, Facultad de Química, Universidad de la Habana, La Habana, Cuba; ^3^ School of Biological Sciences and Engineering, Yachay Tech University, Urcuquí, Ecuador; ^4^ Department of Surfactants and Nanobiotechnology, Institute for Advanced Chemistry of Catalonia (IQAC-CSIC), Barcelona, Spain; ^5^ CIBER-BBN, Networking Centre of Bioengineering, Biomaterials, and Nanomedicine and Department of Organic Chemistry, University of Barcelona, Barcelona, Spain; ^6^ School of Chemistry and Physics, University of KwaZulu-Natal, Durban, South Africa

**Keywords:** drug delivery systems, biomolecules, carriers, covalent bioconjugation, linkers

## Abstract

Cancer is one of the diseases with the highest mortality rate. Treatments to mitigate cancer are usually so intense and invasive that they weaken the patient to cure as dangerous as the own disease. From some time ago until today, to reduce resistance generated by the constant administration of the drug and improve its pharmacokinetics, scientists have been developing drug delivery system (DDS) technology. DDS platforms aim to maximize the drugs’ effectiveness by directing them to reach the affected area by the disease and, therefore, reduce the potential side effects. Erythrocytes, antibodies, and nanoparticles have been used as carriers. Eleven antibody–drug conjugates (ADCs) involving covalent linkage has been commercialized as a promising cancer treatment in the last years. This review describes the general features and applications of DDS focused on the covalent conjugation system that binds the antibody carrier to the cytotoxic drug.

## 1 Introduction

The drug delivery system (DDS) is a strategy used to improve the target delivery or to control the release of pharmaceutical products in patients (Vega-Vásquez et al., 2020; Jeong et al., 2021a), which has been broadly accepted in the pharmaceutical industry. During the design and development of DDS, two main features are needed: drug targeting and controlled release (Volpi et al., 2021). The first is related to the area where the expected drug arrives, and the second feature is related to two variables: rate and time of drug release. In other words, a DDS is defined by the rate and the time it takes to release the drug in a specific area (Ojediran and Raji, 2010; Jeong et al., 2021b).

Regarding DDS development, the oral or intravenous route has been the most traditional drug delivery administration (Ojediran and Raji, 2010; Briolay et al., 2021), although others, including intranasal administration have also been developed in the last few years (Zhang et al., 2021c).

The main goal of DDS is to reduce the side effects usually caused by treatments and, therefore, allow the use of even more toxic drugs and improve the chemical stability of the drugs. Although cancer has traditionally been the target for DDS, the advent of the new era of oligonucleotide-based drugs is rapidly extending the use of DDS for treating other medical targets. Current cancer treatments are usually not very specific, affecting organs and tissues damage and the healthy others that weaken the patient so that cure can sometimes be as dangerous as the disease. DDS tries to maximize the drugs’ effectiveness by aiming them to a specific area affected by the disease (Xue et al., 2021).

**FIGURE 1:**
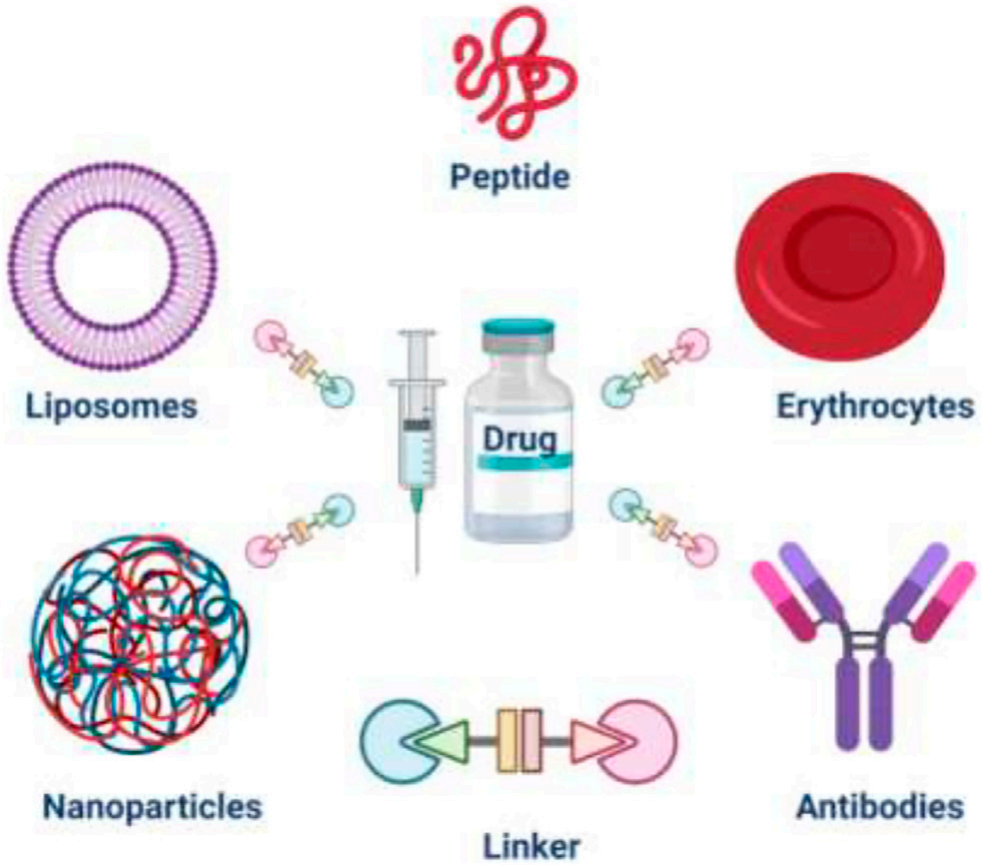
Drug delivery system (DDS) components.

In this context, novel techniques based on chemical conjugations have been carried out to find selective and effective DDS. Recently, a broad range of hybrid covalent entities based on biomolecules and nanoparticles covalently linked to the drug have been developed as efficient drug carriers (Figure 1). Some of them, precisely eleven ADC’s have been commercialized. The present review focuses on those systems designed and prepared through the chemical conjugation of drugs with specific carriers. The fundamental approach to DDS obtained through chemical conjugation using erythrocytes, antibodies, and nanoparticles in the last years are summarized. The main features and their efficiency as therapeutic agents are discussed, focusing on commercialized ADC entities.

## 2 Drugs Delivery Systems Based on Chemical Conjugation

DDS has been developed to find effective and selective entities to counteract several human diseases and avoid or minimize side effects. Bioentities such as erythrocytes or antibodies have been used as biocarriers. On the other hand, nanoparticles as a conveyor in DDS are also summarized (Figure 1).

### 2.1 Erythrocytes (Red Blood Cell) as a Carrier in Drug Delivery Systems

Blood is one of the most critical components of the circulatory system. It is responsible for irrigating the entire human body, and erythrocytes, also called red blood cell (RBC), are the most numerous cells present in the blood. RBC transport and exchanges oxygen and carbon dioxide between the lungs and tissues of other organs (Weisel and Litvinov, 2019). Erythrocytes are biocompatible, biodegradable, and nonimmunogenic, making them enjoyable to use in DDS development (Glassman et al., 2020; Zhang X. et al., 2021). The first report of DDS based on erythrocytes described the Daunorubicin linked through a chemical bond to nanoerythrocytes using glutaraldehyde as a homodifunctionalized linking arm. It was observed that the cytotoxicity of the conjugated drug was higher than the free drug when assessed on the P388D1 cancer cell line (Lejeune et al., 1994).

Erythrocytes are being successfully used in some pharmacological therapies due to their potential to transport molecular cargos in three different ways, inside it (embedded), physically adsorb nanocarriers onto its surface (RBC hitchhiking), or chemically linked to drugs (Villa et al., 2017; Alapan et al., 2018; Luo et al., 2021). We highlighted the third way in which chemical conjugation is present.

**FIGURE 2:**
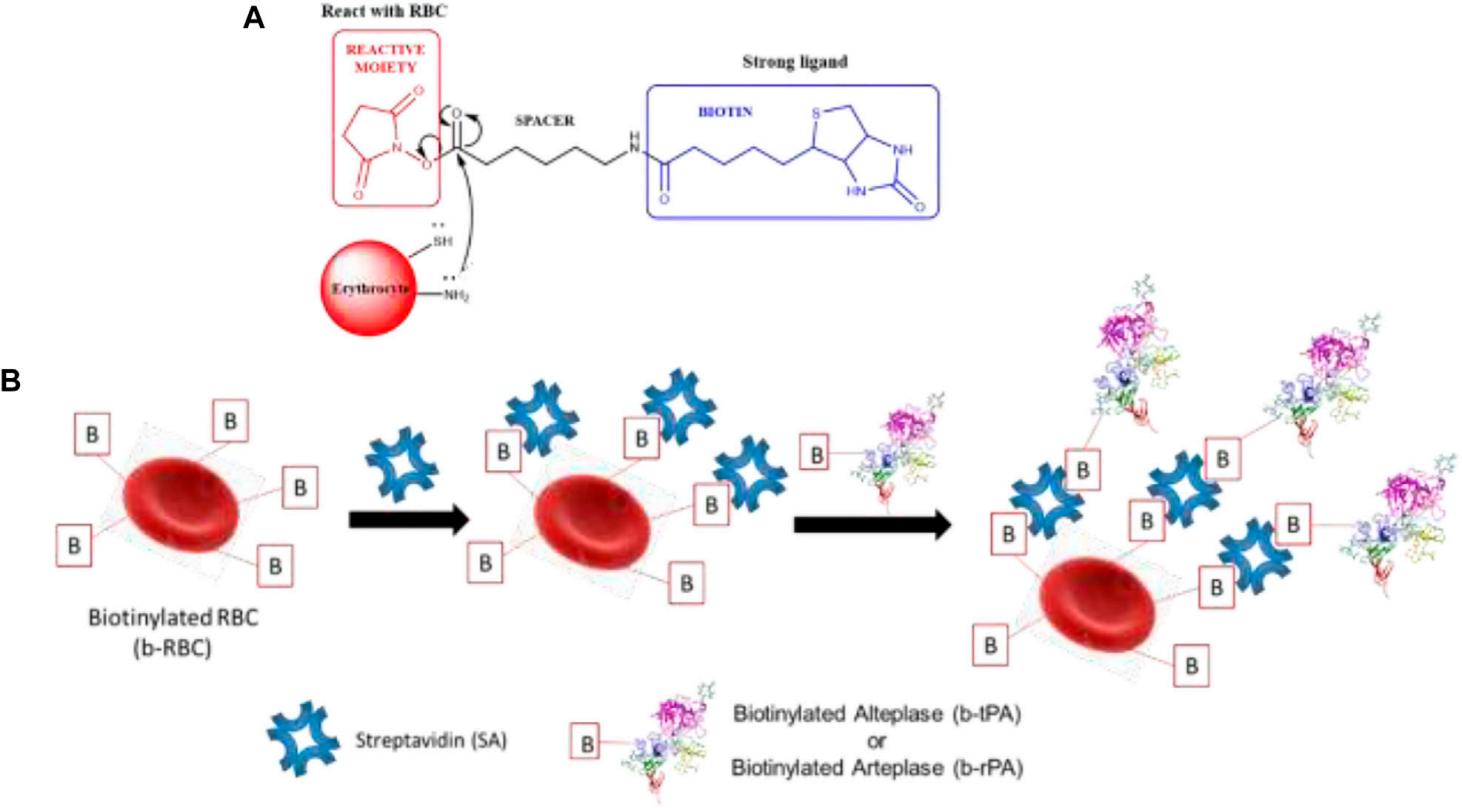
**(A)** Biotinylation reagents general structure. The reagent shown is NHS-LC-biotin, **(B)** a streptavidin-biotin technique using RBC as a carrier.

Ganguly et al. reported erythrocytes and tissue plasminogen activator (tPA or rPA) conjugation through a streptavidin-biotin technique in thromboprophylaxis. Before conjugation between erythrocytes and tPA/rPA, biotinylation of both biomolecules was carried out (Ganguly et al., 2005; Ganguly et al., 2007). Biotinylation is most commonly performed through chemical means, although enzymatic methods are also used. Nowadays, many biotinylation reagents are available, so chemical methods provide greater flexibility in the type of biotinylation needed. All biotinylation reagents have similar features, where the biotin group is present, but a reactive moiety gives biotinylation reagents distinct characteristics that are ideal for different types of experiments (Figure 2A) (Halfon-Domenech et al., 2011; Samavarchi-Tehrani et al., 2020). Because biotin has an extremely high affinity to the protein streptavidin, biotinylated erythrocytes (b-RBC) join streptavidin (SA) noncovalently, and the hybrid obtained entity is then conjugated to biotinylated alteplase (b-tPA) or alteplase (b-rPA) (Figure 2B). The final conjugate made it possible to improve the resistance of tPA to PAI-1 inhibitors, and hence these have a longer intravascular life (Ganguly et al., 2007). The conjugation of urokinase plasminogen activator (scuPA) to RBC as a carrier has also been reported using the same avidin-biotin technique (Murciano et al., 2009). The purpose of the conjugation of both receptors (b-RBC and b-tPA/b-rPA) is to prevent blood coagulation called thromboprophylaxis.

In the same way, a multifunctional DDS based on RBCs for magnetic field-enhanced drug delivery and imaging-guided combination therapy of cancer has been described (Wang et al., 2014). Biotin was covalently linked to RBC, and iron oxide nanoparticles (IONPs) coated with chlorine e6 (Ce6), a clinically used photodynamic agent, were attached to the membrane of the RBCs through avidin-biotin binding. Then, doxorubicin (DOX), a known anticancer drug, was loaded inside the RBCs, obtaining a DOX@RBC-IONP-Ce6-PEG structure with a long blood-circulation time and robust responses to the external magnetic field (Wang et al., 2014).

**FIGURE 3:**
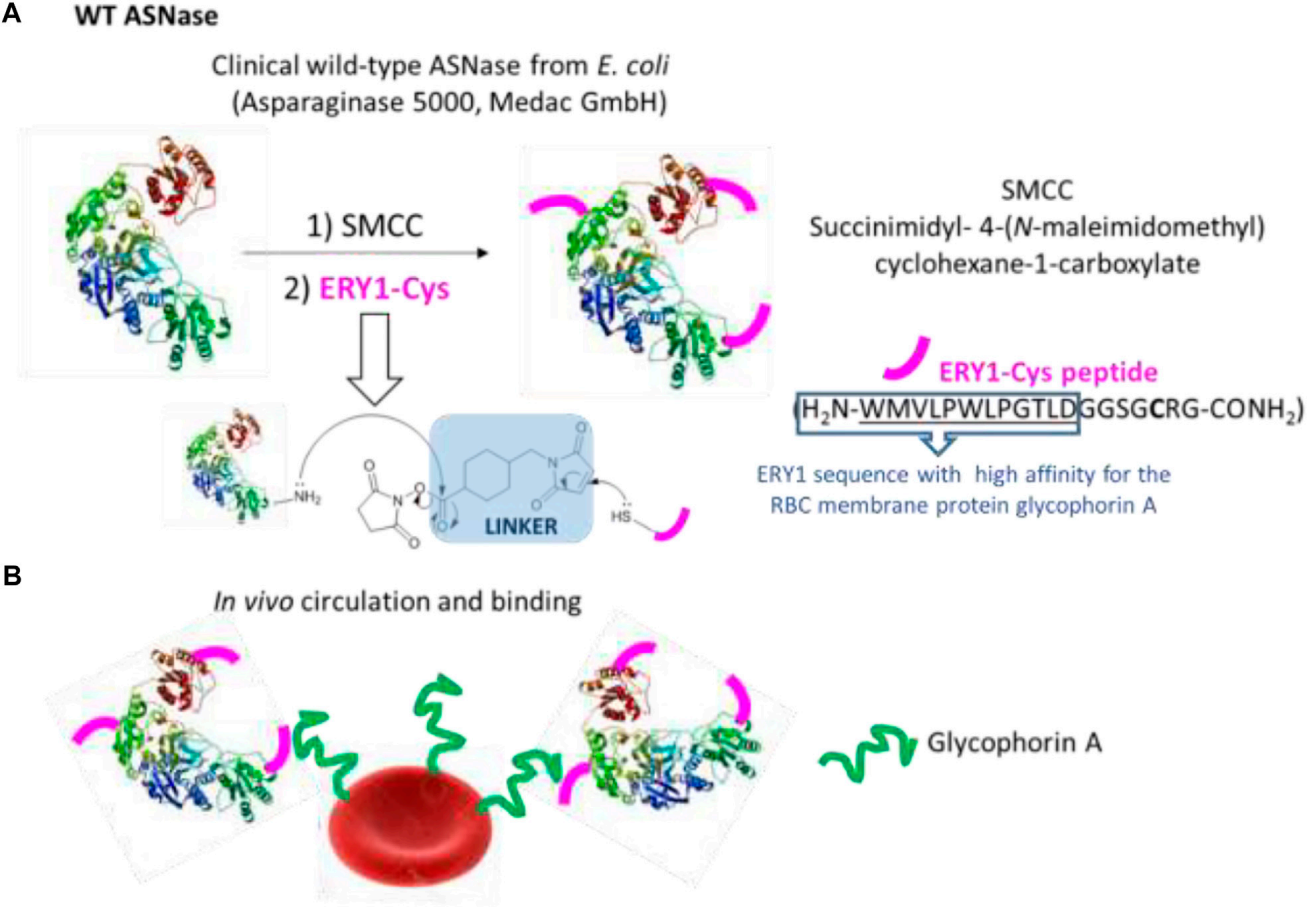
Conjugation of *Escherichia coli* L-asparaginase-II (ASNase) with copies of glycophorin A-binding peptide (ERY1). **(A)** Chemical conjugation between Lys residues of ASNase and peptide ERY1 using SMCC as a linker, **(B)** high-affinity binding of ASNase to erythrocytes through chemical conjugation of several copies of glycophorin A–binding peptide (ERY1).

Another example of erythrocyte use in DDS was reported by Lorentz et al. (2015), which described the conjugation of *Escherichia coli* L-asparaginase-II (ASNase) with some copies of glycophorin A-binding peptide (ERY1). The procedure started with the chemical conjugation between Lys residues of ASNase and peptide ERY1 using succinimidyl 4-(N-maleimidomethyl)cyclohexane-1-carboxylate (SMCC) as a linker (Figure 3A). SMCC is one of the most useful bifunctional linkers used in bioconjugation, allowing the first reaction *via* amide formation (free amines from biomolecule or cargo and succinimidyl ester from SMCC). Then, the maleimide moiety would react with sulfhydryl groups of another entity (biomolecule or payload). After that, high-affinity binding of ASNase to erythrocytes was achieved through chemical conjugation of several copies of glycophorin A–binding peptide (ERY1). This peptide (ERY1) was conjugated to ASNase through its cysteine residue (Lorentz et al., 2015) (Figure 3B).

The conjugation described earlier reduces the development of antibodies because it induces antigen-specific tolerance. Erythrocytes conjugated with ASNase have a drug pharmacodynamics effect more significant than ASNase administrated alone. Grimm et al. (2015b) used the same ERY1 peptide chemically conjugated the erythrocyte to ovalbumin through sulfosuccinimidyl-4-(N-maleimidomethyl)cyclohexane-1-carboxylate (Sulfo-SMCC) as a linker. This conjugation improved the response of CD4^+^ and CD8^+^ T cells to an antigen because these induced an antigen tolerance (Grimm et al., 2015a; Glassman et al., 2020).

#### 2.1.1 Erythrocytes Based Drug Delivery Systems and the Market

Nowadays, there are no FDA-approved conjugate erythrocytes, but there are studies related to drugs encapsulated in erythrocytes in phases I, II, and III, such as those related to the dexamethasone-21-phosphate in ataxia-telangiectasia, asparaginase in pancreatic cancer, lymphoblastic leukemia, thymidine phosphorylase in mitochondrial neurogastrointestinal encephalomyopathy and RTX-134 in phenylketonuria (Rossi et al., 2020; Javed et al., 2021). In clinical trials, none of the conjugates include covalent bond formation in their preparation. The studies bet on the encapsulation of the drug or the mixture of drugs, using the erythrocytes as a carrier (NCT03267030, 2021; NCT02195180, 2014; Hammel et al., 2020; Rossi et al., 2020).

The DDS based on erythrocytes is complicated to bring to market because it needs to fulfill a pharmaceutical product’s parameters. Among specifications that make complex their market development are: 1) cell material source, which refers to the erythrocytes that come from analogous or homologous donated blood; 2) manufacturing process, which refers to the sterility of the cell suspension and the reduction of leukocytes; 3) product storage, regarding the solution where the erythrocytes are reserved to their prompt administration because they tend to degrade, as well as the problems associated with prolonged release of the cargo, and the potential interactions between cargo and erythrocytes (Bourgeaux et al., 2016; Rossi et al., 2020). Probably, erythrocytes-based DDS will be approved by the Center for Biologics Evaluation and Research (CBER) as the blood and other similar derivatives.

### 2.2 Nanoparticles as the Carrier in Drug Delivery Systems

Nanoparticles (NPs) are entities with an ultrasmall size; generally, with dimensions less than 200 nm, these dimensions confer different physical, chemical, or/and biological properties compared to a macro or microsample for the same material (Lungu et al., 2015; the United States Environmental Protection Agency, 2021). Nanoparticles could be classified as inorganic and organic NPs, where the first one involves gold and silica NPs, and the second are related to liposomes, micelles, and polymeric NPs (De Matteis and Rinaldi, 2018).

Some valuable features of NPs related to DDS are 1) suitable to be used in intravenous delivery, 2) a good behavior as site-specific drug targeting to treat several diseases, 3) a higher surface to volume ratio as compared with bulk material would provide a diminution in the dose of the drug and therefore a reduction in the toxicity; and 4) a prolonged circulation in the blood among others, which make them suitable for its use as DDS and promising results had been reported (Werengowska-Ciećwierz et al., 2015; Prasher et al., 2020; Cevaal et al., 2021).

Drugs can generally be aggregated on the external or internal nanocarrier surface or linked through chemical bioconjugation. Focused on DDS based on nanoparticles, emphasizing chemical conjugation, different kinds of NPs as polymeric (i.e., PLGA, PLA, PEG, hydrogel, chitosan analog), magnetic, and structures based on carbon have been extensively described (Friedman et al., 2013; Saw et al., 2021; Zielińska et al., 2021).

**FIGURE 4:**
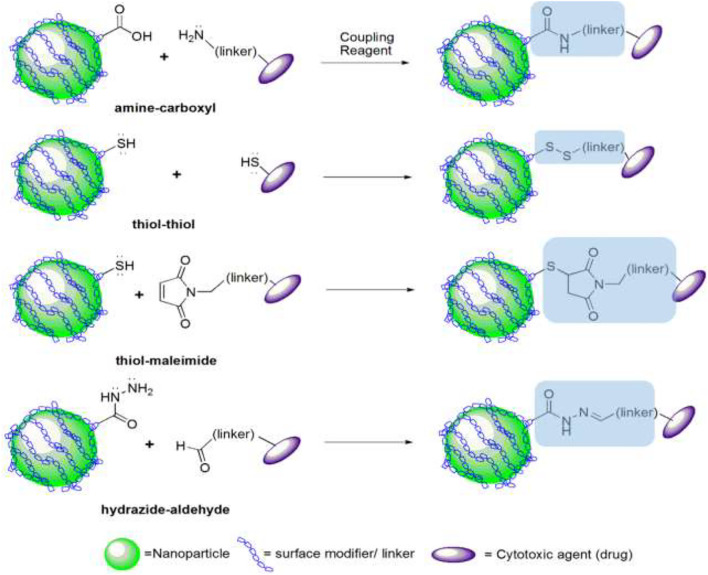
Strategies of conjugation for DDS based on the modified nanoparticle surface.

Chemical conjugation usually takes place on the surface of NPs. Different strategies are used to conjugate NPs with drug-like reactions. Amine-carboxyl, thiol-maleimide, thiol-thiol, hydrazide-aldehyde, and gold-thiol are the most priceless reactions used in the conjugation with NPs, allowed to generate amide, thioether, disulfide, hydrazine, gold-thiol, and triazole ring as covalent linkage, respectively (Friedman et al., 2013; Nikezić et al., 2020). In this regard, the drug or ligand structure determines the type of reaction (Figure 4).

#### 2.2.1 Nanoparticles Based on Liposomes as the Carrier in Drug Delivery Systems

NPs based on liposomes can be considered one of the most hopeful carriers when incorporated into a controlled drug release system (DDS).

**FIGURE 5:**
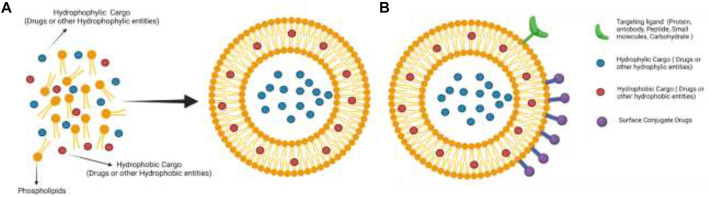
**(A)** General liposome formation. **(B)** The general structure of DDS is based on functionalized liposomes.

Liposomes are spherical vesicles that can be made from a suspension of phospholipids in a hydrophobic medium; this solvent is slowly removed or replaced by another hydrophilic, usually an aqueous solution, using dilution, evaporation, or dialysis processes. This change of solvent causes the phospholipids to self-assemble spontaneously. In this way, they form spherical bilayers and trap a large amount of the aqueous medium in which they are found. They vary in size but are generally smaller than 400 nm. When working with drugs or hydrophilic entities, they are trapped inside the bilayer; and the hydrophobic remains in the phospholipid layer (Figure 5A) (Akbarzadeh et al., 2013; Craciunescu et al., 2021; Kanojia et al., 2022).

This carrier is one of the most studied to be used in DDS due to liposomes’ positive features. For example, they have a high loading level and a long time in circulation, changing drug pharmacokinetics compared to the drug administered alone. Liposomes as carriers are safe, biodegradable, and biocompatible, making them beneficial to theragnostic (Sonali et al., 2016) (Alwattar et al., 2021) even with imaging purposes when these are conjugated with quantum dots or gold nanoclusters (Liao et al., 2021; Yao et al., 2021).

In general, liposomes can transport drugs in different ways: trapped inside the bilayer (hydrophilic drug), remain in the phospholipid layer for hydrophobic drugs, and covalently link to the liposome surface (Figure 5B). Regarding chemical conjugation, ester and amide bonds have been extensively used in two ways: 1) to join phospholipid to the drug and then carry out the liposome formation; and 2) to decorate the liposome surface.

Liposomes have been used as biocarriers, and their conjugation with microbubbles (Chandan and Banerjee, 2018), some proteins (Sonali et al., 2016; Wang et al., 2021), or antibodies (Burande et al., 2020) allowed to obtain even DDS with anticancer activity (Zhang Y. et al., 2021). Different chemical approaches with different functional groups are used to carry out the conjugation (Aguilar-Pérez et al., 2020), but the more used are ester bonds that are formed between a carboxylic acid group and a hydroxyl group that could have the drug and the lipid, respectively. A linker or spacer such as succinic acid is used to facilitate this conjugation (Irby et al., 2017; Palko-Łabuz et al., 2021). Another helpful linker to lead the conjugation is a succinic anhydride moiety due to being functionalized (Muthu et al., 2015; Spanedda and Bourel-Bonnet, 2021).

Amide bonds are generated between a terminal carboxylic acid of the lipid and an amine group present in the drug (Lanigan et al., 2016; Papadopoulos and Kokotos, 2016; Sharma et al., 2021).

NPs have been conjugated to improve the pharmacokinetic and pharmacodynamic properties of these cargos, overcoming inconveniences like multidrug resistance (Nikezić et al., 2020).

#### 2.2.2 Drug Delivery Systems Based on Nanoparticles as Marketed Drugs

**FIGURE 6:**
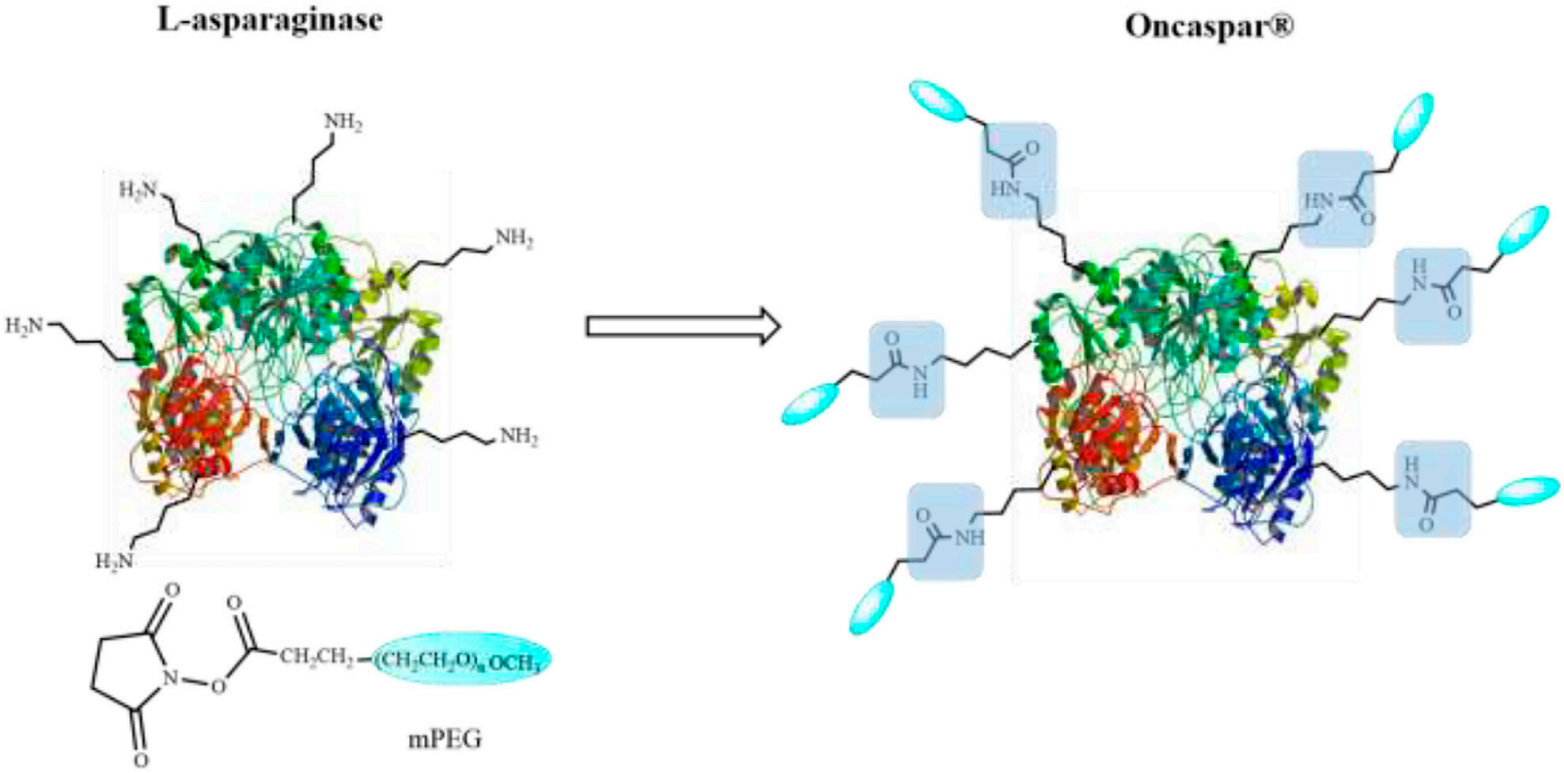
Pegylation of L-asparaginase to obtain a nanotherapeutic product Oncaspar^®^.

Nowadays, in nanomedicine, PEGylated polymers nanoparticles are the most used and the most available in the market. For instance, Oncaspar^®^ was one of the first nanotherapeutic approved by the FDA in 1994 (U.S. Food and Drug Admins, 1994), formed by L-asparaginase aminohydrolase covalently conjugated to monomethoxypolyethylene glycol (mPEG) through nonspecific random PEGylation of ε-amino groups on the lysine residues of the enzyme (Figure 6), which is used in chemotherapy for the treatment of acute lymphoblastic leukemia (ALL) (European Medicines Agency). Also using PEGylation procedures, Krystexxa^®^ was developed in 2010 (U.S. Food and Drug Admins, 2010). The NPs consisted of a pegloticase and mPEG covalent conjugate and were designed to treat severe debilitating chronic tophaceous gout (European Medicines Agency, 2013). Four years later, Plegridy^®^ was introduced in the pharmaceutical market (U.S. Food and Drug Admins, 2015) to treat relapsing-remitting multiple sclerosis (European Medicines Agency). PEGylation of interferon beta-1a using mPEG and O-2-methylpropionaldehyde linker was reported as its formation reaction of NPs (U.S. Food and Drug Admins, 2015) (European Medicines Agency). The last polymer-drug conjugated approved was ADYNOVATE (Amin, 2008) in 2015, which is a covalently conjugated ADVATE molecule with PEG. This conjugate is used to treat and control hemophilia A (RxList).

Several of these pharmaceutical products with novel technologies attract the research community’s attention. Invega Trinza^®^ (Daghistani and Rey, 2016), with nanocrystal technology based on Paliperidone Palmitate structure, releases paliperidone slowly over a long period for the treatment of schizophrenia and schizoaffective disorder. Sublocade^®^ (buprenorphine extended-release) (Daghistani and Rey, 2016) with the Atrigel technology *in situ* gel-forming system polymer (biodegradable 50:50 poly(DL-lactide-co-glycolide), and a biocompatible solvent, N-methyl-2-pyrrolidone (NMP) is thought to be a revolutionary product administrated to treat moderate to severe opioid use disorder. The most critical Sublocade^®^ disadvantage is that it can only be administered *via* subcutaneous. In fact, serious harm or even death would result if this drug is administered intravenously.

DDS based on liposomes approved by the FDA are scarce, and most of them are PEGylated liposomes in which there is no covalent conjugation. For instance, the antibiotic doxorubicin used in chemotherapy was combined with liposome conjugated with PEG (Doxil^®^) to improve the circulation time of the drug and reduce toxicity (Gabizon, 2001). It was the first DDS based on liposomes approved by the FDA in 1995 (U.S. Food and Drug Admins, 1995). Twelve years later, Liposomal doxorubicin, in combination with another anticancer drug, bortezomib (Lipodox^®^), was developed for relapsed or refractory multiple myeloma (U.S. Food and Drug Admins, 1995; Ning et al., 2007). Lipodox^®^ has not been approved yet by the FDA, but given the shortage of Doxil, it was accepted for the importation of Lipodox^®^ because it has the same active ingredient, dosage, strength, and route of administration as the aforementioned FDA-approved drug Doxil (U.S. Food and Drug Admins, 2012a).

Additionally, some DDS based on PEGylated liposomes are still in clinical trials, and some good examples are thermosensitive doxorubicin in phase III, which is a promising treatment for liver cancer (Andriyanov et al., 2014; Yang et al., 2019). Liposomal Irinotecan in phase I could be used in advanced refractory solid tumors, advanced esophageal squamous cell carcinoma, and metastatic pancreatic adenocarcinoma (Chang et al., 2015; Bang et al., 2021; Liu et al., 2021).

The nanomedicines Patisiran/ONPATTRO, VYXEOS, and NBTXR3/Hensify have also been approved in the last few years (Anselmo and Mitragotri, 2019). Patisiran/ONPATTRO could be described as RNA-delivering lipid-based nanoparticles, which specifically inhibit hepatic synthesis of transthyretin. It was developed and marketed by Adams et al. (2018). On the other hand, VYXEOS is a liposomal formulation of daunorubicin and cytarabine in a fixed combination for the treatment of adults with newly diagnosed therapy-related acute myeloid leukemia (t-AML) or acute myeloid leukemia (AML) with myelodysplasia-related changes (AML-MRC) (Krauss et al., 2019). Finally, NBTXR3/Hensify is a new class of radio enhancer hafnium oxide nanoparticles sold by Nanobiotix (Bonvalot et al., 2017; Bonvalot et al., 2019).

In the last years, research related to the controlled release of drugs has moved toward the nanoscale. In this sense, several investigations aim to develop robust nanocarriers (nanoliposomes, polymeric, micelles, albumin-bound nanoparticles) with various biomedical, safe, and fast-acting applications (Anselmo and Mitragotri, 2019; Aguilar-Pérez et al., 2020; Tang et al., 2021). Although the most marketed liposome and NPs based DDS does not involve chemical conjugation, the broad range of DDS applying covalent conjugation and included in clinical trials, showed the near future of this field, promoting DDS with good stability, low toxicity, high control of particle size, and modulating the drug release.

### 2.3 Proteins as a Carrier in Drug Delivery Systems

Proteins are high molecular weight biomolecules commonly used to make DDS. Animal-originated proteins such as silk fibroin, collagen, gelatin, and mainly albumin and antibodies are proteic entities widely used for DDS (Jacob et al., 2018).

#### 2.3.1 Albumin as the Carrier in Drug Delivery Systems

Albumin can be considered an excellent drug delivery platform because of its biochemical and biophysical properties. Anti-cancer drugs bound to albumin often benefit from significant advantages, including longer circulatory half-lives, tumor-targeted delivery, and easier administration relative to the drug alone (Sleep, 2015; Xu et al., 2022; Xu et al., 2022). Based on the albumin binding strategy, covalent conjugations are one of the most used methods of drug control release. Standard methods for direct, covalent conjugation involve binding the drug to either lysines, tyrosines, or the free SH-group on the cysteine-34 amino acid residue of albumin. In this regard, Etelcalcetide, a D-amino acid peptide, undergoes reversible disulfide exchange with serum albumin to form a serum albumin peptide conjugate (SAPC) that is too large (67 kDa) to be dialyzed. This disulfide bond between albumin and peptide allowed for a controlled release of Etelcalcetide, improving its pharmacokinetics (Wu et al., 2018).

Stenzel et al. have developed several procedures to apply the conjugation via the covalent bond of albumins with different cargos and promote the nanocarriers formation (Jiang et al., 2014; Dag et al., 2015; Dag et al., 2015; Taguchi et al., 2018). In this sense, albumin was conjugated *via* thiol-maleimide with maleimide functionalized poly[oligo(ethylene glycol) methyl ether methacrylate] to generate a polymer–albumin conjugate, which was able to condense positively charged proteins (Taguchi et al., 2018). In addition, poly(2-dimethylaminoethyl methacrylate was conjugated to albumin as a promising gene carrier (Taguchi et al., 2018).

(6-Maleimidocaproyl)hydrazone derivative of doxorubicin (ALDOX) has been conjugated to albumin through an acid hydrolyzable linker, demonstrating *in vitro* activity comparable to free doxorubicin (DOX) in breast and leukemia cell lines. The acid cleavable linker promotes the putative drug release in the acidic lysosome environment (Taguchi et al., 2018). The pirarubicin (4′-*O*-tetrahydropyranyldoxorubicin, THP), a semi-synthetic derivative of DOX has also been covalently conjugated to albumin to develop pH-sensitive conjugates. Using a thiol-maleimide coupling reaction, maleimide hydrazone derivatives of THP were conjugated with poly-thiolated albumin using 2-iminothiolane, via a thiol-maleimide coupling reaction (Tsukigawa et al., 2020).

Recently, a synthetic method to access covalently bound Bis-N-heterocyclic carbene (bis-NHC) gold(I) (Au(I) bis-NHC) with serum albumin adducts have been developed without affecting the metal center active site. The procedure can be described as a post-synthetic amide conjugation that relies on the use of the new Au(I) bisNHC complex bearing a carboxylic acid functional group (Sen et al., 2021).

#### 2.3.2 Antibody as a Carrier in Drug Delivery Systems (Antibody-Drug Conjugates)

An antibody is a Y-shaped protein generated by the immune system in response to antigens such as infectious agents or foreign substances. Their main characteristic is its ability to bind to a specific region or epitope of the corresponding antigen, which allows the antibody to be specific and therefore has demonstrated applications in the treatment of some diseases, i.e., cancer (Whalley et al., 2018). In general, monoclonal antibodies (mAb) are generated through the hybridoma technique (Köhler and Milstein, 1992), which uses murine B cells and murine myeloma cells to produce murine mAbs with a unique specificity. The mAbs have been used to develop bioconjugation techniques, using chimeric and humanized antibodies with therapeutic purposes and hence in bioconjugation (Chiu et al., 2021; Ferraro et al., 2021).

Undoubtedly, one of the most successful DDS therapeutic alternatives is the Antibody-Drug conjugates (ADCs) due to the high specificity of these biomolecules and the structural facility (several functional groups available) to load the drug. For this reason, the amount and concentration of the cytotoxic agent used for the treatment are lower than the treatment of naked antibody or drug only (Diamantis and Banerji, 2016; Ferraro et al., 2021; Khattak et al., 2021). Since the objective is to focus cytotoxicity on tumor cells, patients tend to tolerate ADC treatment for more time. Another advantage of ADCs is that it takes more time to develop resistance than their constituent mAb (Thery et al., 2014; Dhritlahre and Saneja, 2021), which increases the therapeutic window for a more aggressive fight against cancer. In general, an ADC is formed by the mAb and the drug. Both entities are joined through crosslinking achieved with various reactive groups such as amines, thiols, alcohols, or even carboxylic acids present in either mAb or drug. Another successful way to achieve bioconjugation is the use of a linker. The linker can be a homo- or hetero-biofunctionalized molecule, whether the reaction occurs through the same functional group or two different kinds. In typical homodifunctionalized crosslinkers, conjugation occurs through the same functional group (e.g., conjugation between two amines or two sulphydryl groups). On the other hand, conjugation occurs through two different functional groups (e.g., conjugation between an amine and a thiol group (Kostova et al., 2021).

##### 2.3.2.1 Linker Properties

As mentioned before, the drugs could be conjugated directly to the antibody. However, the linkers have helped prepare ADCs to facilitate bioconjugation reactions. Among the advantages of using a linker are preventing premature drug release, improving the liberation of the active drug at the target, and facilitating stability in the production process. The most important aspect of the linker is its physicochemical properties, which allows defining two main different kinds of linker:

Cleavable linker: It is characteristic of breaking one of its chemical bonds that binds the two molecular entities. This kind of linker has been successful in elaborating ADCs due to its stability in the synthesis process and the blood circulation of the conjugate for a long time. It allows the releasing of the drug under some conditions at the surrounding of the target cell or tissue-like overexpression of enzyme or acidic environment (Lu et al., 2016; Ramos Tomillero, 2016; Kostova et al., 2021).

Non-cleavable linker: Its principal function is to maintain links between biomolecule and drug. This feature represents a more significant advantage over cleavable linkers because it has more excellent stability in the plasma, thus increasing the specificity. The drug will not be released outside the target cells because the mAb degradation is carried out within the lysosome (McCombs and Owen, 2015; Ramos Tomillero, 2016; Kostova et al., 2021).

##### 2.3.2.2 Chemical Conjugation

Chemical conjugation in the ADC uses covalent bond formation. This delivery system uses a linker between the cytotoxic agent and antibody (Parslow et al., 2016; Kostova et al., 2021). Preferred active sites on antibodies are the amine group of a lateral chain of exposed lysines or the sulfhydryl group of the exposed cysteines. The covalent coupling can be made mainly through two methods: lysine to amide or cysteine to nucleophilic attack.

Lys amide coupling: this method binds payload and Lys residues on the antibody using linkers containing activated carboxylic acid active esters. However, this conjugation can give multiple ADC species different drug-to-antibody ratios (DAR). With a DAR around of 3-4 because a specific antibody has an average of 10 residues of Lys chemically available of around 80 Lys that it would have. Therefore, this way of chemical conjugation generates a heterogeneous mixture with some species challenging to characterize and purify, which could provoke effects on the binding to the antigen and even on the safety profile of the ADC (Parslow et al., 2016; Tsuchikama and An, 2018).

Cys nucleophilic attack: this method binds payload previously modified with the thiol-reactive group and Cys residues of the antibody, generally using maleimide-type linkers (Parslow et al., 2016; Tsuchikama and An, 2018). Cys are less frequent in Abs than Lys, and there are forming disulfide bridges that are key for the structure of the Abs. Thus, the first step in using Cys as a reactive moiety of the Abs is to reduce the disulfide bridges partially. This step is critical because overreduction will lead to the Abs’ denaturalization. For example, in the antibody IgG type (commonly used in ADC), 16 Cys are present: twelve intrachain and four interchain disulfide bonds. These four interchain disulfide bonds are the target for conjugation because these are not important for structural stability (Zakharov et al., 2000), allowing limited conjugation sites (2-8 free thiols) due to the reactivity of the thiol group. There is still the possibility to end up with a heterogeneous mixture of ADC, although to a less extent than Lys conjugation. Cys coupling has been mostly used in clinical trials because it has a higher DAR (approximately between 0 and 8) than Lys amide coupling. The conjugates obtained with this technique are easier to characterize (McCombs and Owen, 2015; Tsuchikama and An, 2018).

Regarding cancer disease, the significant advantage of these systems is the delivery of the drug, which could have high toxicity, directly into the tumor (Goss et al., 2018; Dhritlahre and Saneja, 2021; Ferraro et al., 2021). The possibility to combine the favorable binding properties of mAbs with the activity of potent cytotoxic agents promises to increase the therapeutic index of therapeutic payloads.

**FIGURE 7:**
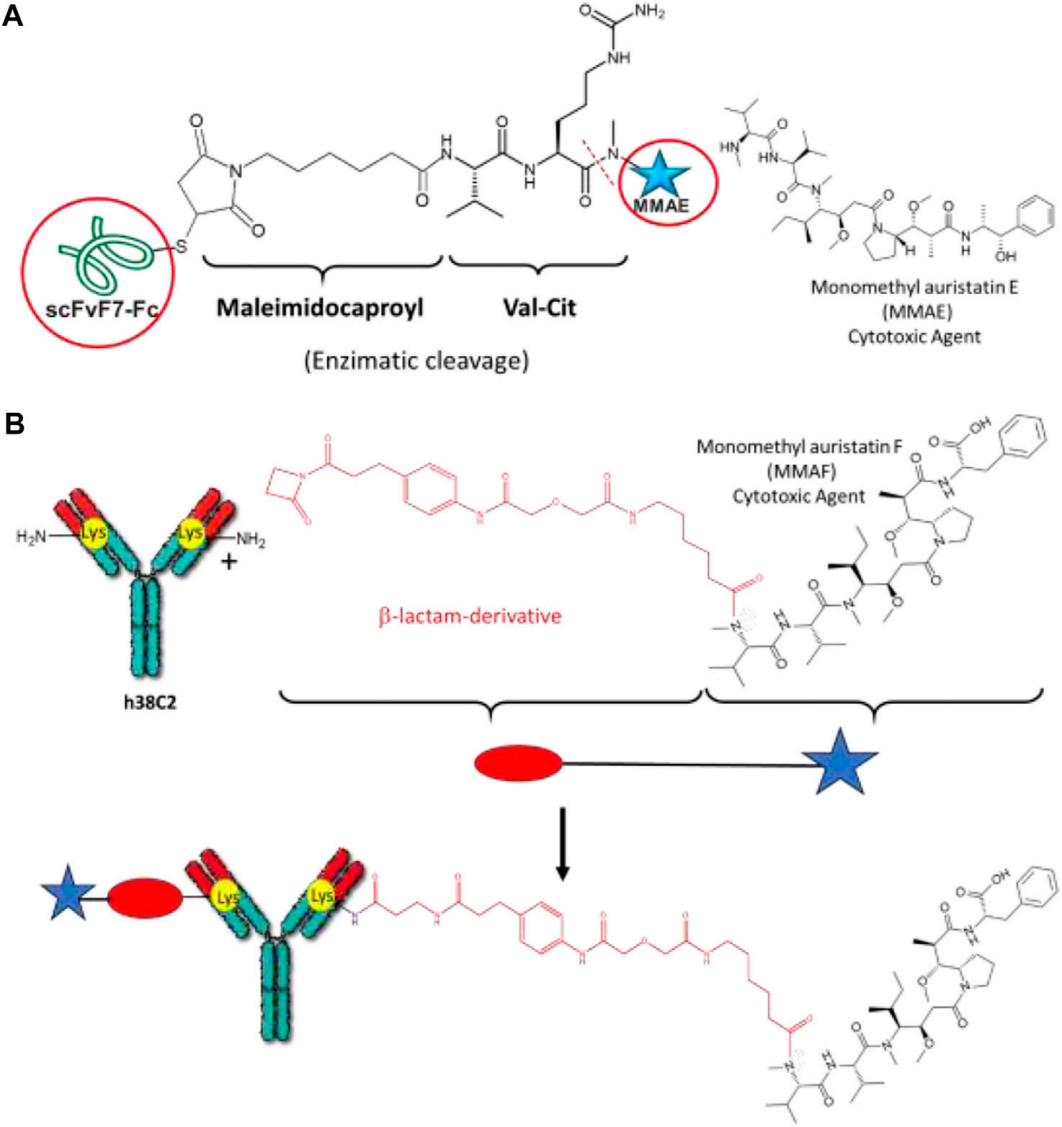
**(A)** Structures of the drug (MMAE) and mAb-drug conjugate. **(B)** Conjugation of the humanized anti-hapten monoclonal antibody (mAb) h38C2 through nucleophilic lysine with β-lactam derivatives (i.e., β-lactam functionalized monomethyl auristatin F (MMAF).

Borek et al. (2018) reported a chemical conjugation of scFvF7-Fc antibody fragment through its cysteine residues to cytotoxic agent monomethyl auristatin E (MMAE) using a valine-citrulline linker and a maleimidocaproyl spacer (Doronina et al., 2003). In this case, enzymatic and selective cleave occurs in the bond between the peptide with the termini valine-citrulline and the corresponding drug (Figure 7A). This ADC can be internalized into cancer cells due to its composition, i.e., the antibody and linker. This study was carried out in search of an alternative for treating gastrointestinal cancer, with a high incidence of mortality and relatively short survival rates.

Recently, biophysical characterization of a DAR 8 ADC fully conjugated at all interchain Cys residues was reported to keep the structure and stability of the fully conjugated as an optimized and clinically relevant second-generation monomethyl auristatin-E (MMAE) drug-linker (Chiu et al., 2021).

On the other hand, numerous studies using Lys residues of ADCs have been carried out. In general, Lys-mediated chemical bioconjugation allows to obtain heterogeneous conjugates mixtures with 0–8 DAR, and consequently, each of them has different toxicity, pharmacokinetics, and efficacy. Although both Cys and Lys-assisted conjugation is still used, some alternative strategies have also been implemented to generate more homogeneous ADCs. For example, Nanna et al. (2017) reported the conjugation of the humanized antihapten monoclonal antibody (mAb) h38C2 through its most exceptionally nucleophilic Lys present at an 11-Å deep hydrophobic pocket in their structure. The mentioned Lys is capable of catalyzing aldol and retroaldol reactions. Thus, it can be selectively conjugated with β-lactam derivatives (i.e., β-lactam functionalized monomethyl auristatin F (MMAF)), generating a noncleavable and stable amide bond until its enzymatic degradation inside the target cells takes place (Figure 7B). The described conjugate could avoid the premature release of the cytotoxic agent. The other achievement of this study is generated homogeneous ADC’s (Nanna et al., 2017).

Valine-citrulline dipeptide has been used as a linker for ADCs such as DLYE5953A, a humanized IgG1 monoclonal antibody that targets the lymphocyte antigen 6 complex (LY6E) conjugated to Monomethylauristatin (MMAE) under the auspices of Genentech and showed antitumor activity in patients with solid metastatic tumors (Tolaney et al., 2021).

In the last years, various reports related to multiloading linkers have proposed a novel methodology incorporating two payload molecules into single monoclonal antibodies (mAbs). The main idea is to generate bi or tri-functionalized linkers, in which orthogonal reactions allow the incorporation of different payloads using covalent bonds (Levengood et al., 2017; Kumar et al., 2018; Zhang L. et al., 2021; Yamazaki et al., 2021). Levengood et al. (2017) prepared a linker with sequential Cys residues with orthogonal protection to enable site-specific conjugation of each drug and conjugated to IgG1 through the reduced interchain disulfides of the antibody and stable maleimide linkage. In 2018, a novel heterotrifunctional linker was developed by Kumar et al. (2018), providing a flexible platform that allows the coupling of two different drugs via copper-catalyzed azide-alkyne cycloaddition (CuAAC) and *via* oxime linkage using ketone as starting functional group, but also the conjugation to the antibody through cysteine—maleimide reaction. Recently, a one-pot, successive reaction method to produce dual payload conjugates with the site-specifically engineered cysteine and p-acetyl-phenylalanine using Herceptin (trastuzumab), an anti-HER2 antibodydrug widely used for breast cancer treatment, as a tool molecule as another approach to the specific conjugation of two different drugs, improving not only the potential to overcome drug resistance but enhance the therapeutic efficacy (Zhang L. et al., 2021).

##### 2.3.2.3 Drug Delivery Systems Based on Antibody-Drug Conjugates as Marketed Drugs

**FIGURE 8:**
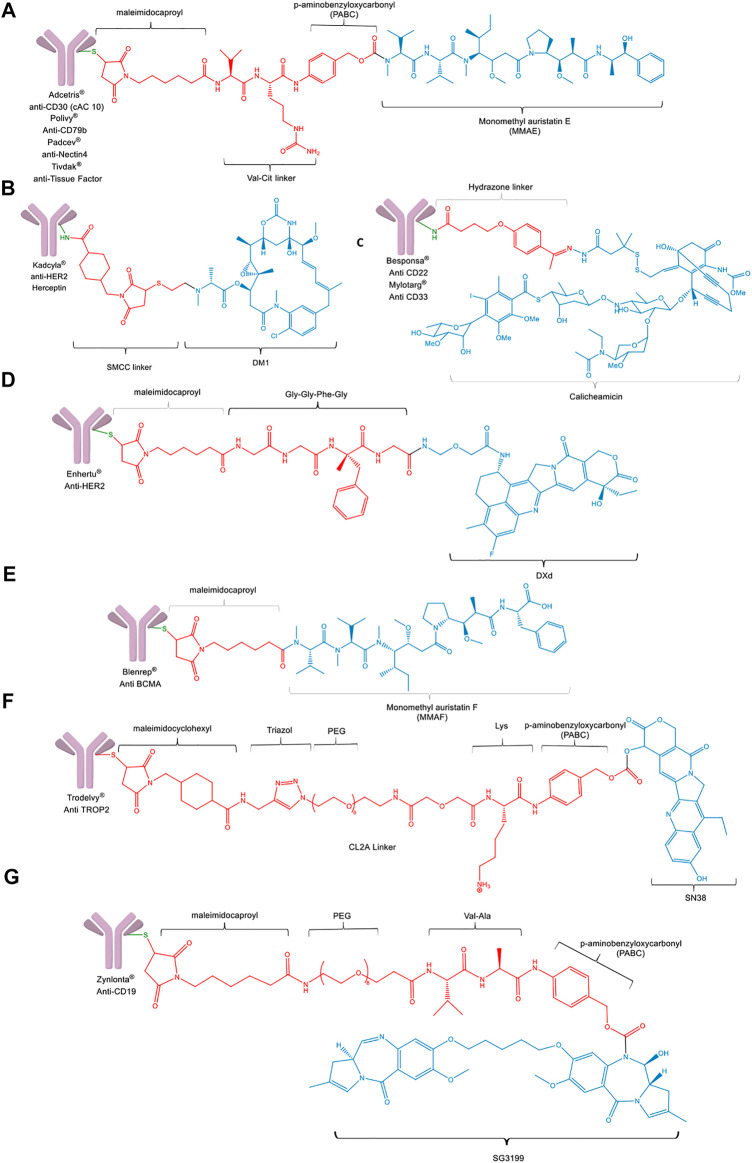
General structures of ADC’ are approved by the FDA. **(A)** Adcetris^®^, Polivy^®^, Padcev^®^, and Tivdak^®^. **(B)** Kadcyla^®^. **(C)** Besponsa^®^ and Mylotarg^®^. **(D)** Enhertu^®^. **(E)** Blenrep^®^. **(F)** Trodelvy^®^. **(G)** Zynlonta^®^. All linkers are highlighted in red.

At present, eleven ADC’s involving chemical conjugation hold market authorization for therapy of certain types of cancers (Figure 8) (do Pazo et al., 2021). Adcetris^®^ (Brentuximab Vedotin, 2013; U.S. Food and Drug Administration (FDA), 2012b) is an antineoplastic agent used in the treatment of Hodgkin lymphoma and systemic anaplastic large cell lymphoma. B.V. is obtained through the covalent conjugation of the mouse-human chimeric monoclonal antibody IgG1 antiCD30 (cAC 10), with monomethyl auristatin E (MMAE). This ADC uses a maleimidocaproyl-valine-citruline-p-aminobenzyloxycarbonyl linker to promote the proteolytic cleavage by Cathepsin B. Through cysteine-maleimide reaction, the corresponding mAb is joined to the potent anti-mitotic agent (monomethyl auristatin E [MMAE]) (Figure 8A). This toxin is a synthetic antitubulin analog, using an enzymatically cleavable dipeptide linker. On average, each antibody molecule was conjugated into four groups of MMAE.

On the other hand, Kadcyla^®^ (Trastuzumab emtansine) (U.S. Food and Drug Administration (FDA), 2013) is an innovative, unique, and selective antineoplastic drug used in patients with advanced breast cancer (HER2+). This ADC is composed of the antiHER2 antibody trastuzumab (Herceptin^®^) and the cytotoxic microtubules agent, DM1, bound through a bifunctional succinimidyl 4-(N-maleimidomethyl)cyclohexane-1-carboxylate (SMCC) as a non-cleavable linker (Figure 8B). This drug acts selectively on HER2-positive tumors cells, exercising, on the one hand, the mechanisms of action of the trastuzumab, and on the other hand, the powerful cytotoxic effect of DM1. The main advantage of this ADC is that it has a selective release, allowing it to minimize its side effects compared to other agents for the same pathology (Baron et al., 2015; U.S. Food and Drug Administration (FDA), 2013).

Besponsa^®^ (Inotuzumab ozogamicina) (U.S. Food and Drug Adminstration (FDA), 2017a) is used in adults with acute lymphoblastic leukemia (ALL) whose disease has stopped responding to conventional chemotherapy (recurrence), or never responded to it (refractory). The carrier of Inotuzumab ozogamicina is a monoclonal antibody that attacks the CD22 protein, which is produced in excess on the surface of lymphoblastic leukemia cells. The antibody binds to a calicheamicin compound that kills cancer cells using a pH-sensitive hydrazone linker (Figure 8C). Once the inotuzumab antibody binds to CD22 in the cancer cells, the calicheamicin is released into the cell, where it damages cellular DNA and causes its death (Hamann et al., 2002; U.S. Food and Drug Adminstration (FDA), 2017a).

Mylotarg^®^ (Gemtuzumab ozogamicin) (U.S. Food and Drug Administration (FDA), 2017b) is used for the treatment of acute myeloid leukemia (AML), a bone marrow cancer. Conjugation is similar to Besponsa; however, the different antibody is used. In this case, Inotuzumab ozogamicin consists of a recombinant humanized IgG4 kappa CD22-targeting monoclonal antibody covalently attached to calicheamicin derivative, which is a potent DNA-binding cytotoxic agent (Figure 8C) (Hamann et al., 2002; U.S. Food and Drug Administration (FDA), 2017b). Mylotarg was removed from the market in 2010 due to the high incidence of death in the patients, but it was restituted in 2017 combined with induction chemotherapy.

In 2019, three ADCs were approved by the FDA, Enhertu^®^, Padcev^®^, and Polivy^®^. The first mentioned conjugate is indicated for the treatment of breast cancer that is metastatic (that has spread to other parts of the body), or that cannot be removed by surgery when cancer overexpresses HER2. The humanized mAb Herceptin (antiHER2) is covalently linked to the topoisomerase I inhibitor deruxtecanan (DXd) using maleimidocaproyl and Gly-Gly-Phe-Gly tetrapeptide as a spacer and protease cleavable linker between the mAb and the cargo (Figure 8D) (Brentuximab Vedotin (SGN35) Drug Description). Padcev^®^ and Polivy^®^ are similar to Adcetris^®^ (Figure 8A) (Process, 2019). Padcev (enfortumab vedotin-ejfv) is an ADC with a synthetic analog of the marine natural peptide dolastatin to treat refractory bladder cancer. In contrast, Polivy^®^ (polatuzumab vedotin-piiq) is indicated to treat adult patients with relapsed or refractory diffuse large B-cell lymphoma.

In 2020, Blenrep^®^ from GlaxoSmithKline and Trodelvy^®^ from Gilead Sciences, Inc. was the ADCs approved by the FDA to treat multiple myeloma and Metastatic triple-negative breast cancer, respectively. Blenrep^®^ includes an antiCD38 monoclonal antibody, a proteasome inhibitor, and an immunomodulatory agent joined by a noncleavable maleimidocaproyl linker (Figure 8E), while Trodelvy^®^ is composed of a humanized monoclonal antibody, hRS7 IgG1κ (sacituzumab), the drug SN-38, a topoisomerase inhibitor maleimidocyclohexyl, and a hydrolyzable linker (called CL2A), which links the humanized monoclonal antibody to SN-38 (Figure 8F) (U.S. Food and Drug Administration (FDA), 2020). The synthetic platform for Blenrep^®^ is similar to those previously described for PADCEV^®^ and Polivy^®^. Nevertheless, the linker of Trodelvy^®^ involves a triazole moiety, PEG units as spacer, and lysine in its structure chemically synthesized (The FDA-Approved Sacituzumab Govitecan for Triple-Negative Breast Cancer | Biopharma PEG).

Last year, Zynlonta^®^ and Tivdak^®^ were incorporated into the list of conjugates FDA approved (Zynlonta FDA. Initial U.S. Approval: 2021, 2021; Tivdak FDA. Initial U.S. Approval: 2021, 2021). Zynlonta^®^ comprises a humanized mAb Anti CD19 conjugated to SG3199, a cytotoxic pyrrolobenzodiazepine (PBD) dimer alkylating agent, through a protease-cleavable valinealanine linker, and using maleimido caproyl and eight PEG units (Figure 8G). This ADC is used for adult patients with relapsed or refractory large B-cell lymphoma after two or more lines of systemic therapy, including diffuse large B-cell lymphoma (DLBCL), not otherwise specified DLBCL arising from low-grade lymphoma, and high-grade B-cell lymphoma (Caimi et al., 2021). Tivdak^®^ shares the synthetic platform as Adcetris^®^, Polivy^®^, and Padcev^®^ (Figure 8A) but uses an antitissue factor as mAb, becoming the first approved ADC indicated for the treatment of adult patients with recurrent or metastatic cervical cancer with disease progression on or after chemotherapy (De Goeij et al., 2015; Coleman et al., 2021).

From the analysis of the recent literature (Tong et al., 2021), it is evident that, referring to controlled drug release systems involving chemical-assisted conjugation, the most successful to date have been the ADCs. Despite the broad range of chemical strategies developed to improve the payload and the ADC’s effectiveness, the most extended is those involving cysteine mediate maleimide conjugation with different linkers highlighting the valine—citrulline, which allows the protease cleavage.

### 2.4 Hybrid Conjugates as Carriers in Drug Delivery Systems

DDS generally uses only one biomolecule or nanoparticle as a carrier, which is sometimes ineffective. Scientists are conducting various experiments where a combination of biomolecules or biomolecules with nanoparticles has been made to find novel hybrid systems for therapeutic use in front of a particular disease, especially cancer. Considering that this disease has various types, it is impossible to have “general” DDS to treat it.

For example, in search of a conjugation brain delivery system able to cross the blood-brain barrier (BBB), Huang et al. (2013) studied lactoferrin (Lf) protein-like active principle conjugated to PEGylated liposomes (PL). The hybrid conjugate was prepared through the thiolization of Lf using Traut’s reagent, allowing to have a sulfhydryl group at the N-terminus. At the same time, PL was functionalized with maleimide to promote a subsequent thioether formation between the previously thiolated Lf and maleimide PL derivatives. Pharmacokinetics and pharmacodynamics properties of the conjugate Lf-PL were compared with PL, taking into account that PEGylation is helpful to improve the properties mentioned earlier (Milton Harris et al., 2001). The results showed a greater uptake of the conjugate system, mediated by the Lf receptor present on the surface of the glioblastomas (Arcella et al., 2015).

The research of Lin et al. (2017) presents an attractive DDS base on liposomes and antibodies. It was achieved by generating a half-antibody with free thiol groups available for the subsequent thioether formation. Selected maleimide-pegylated liposomes loaded with Triptolide (TPL), which has a cytotoxic effect of reducing tumor growth, were conjugated with the mAb and tested in lung tumor cells. Considering that carbonic anhydrase enzyme IX is present on the lung tumor cell’s surface, carbonic antianhydrase antibody IX conjugated to the surface of a liposome was used to target tumors in the lungs to improve the selectivity and precision in this type of DDS. Lung cancer is one of the most afflicted in the world population, and it has lower rates of long-term survival (World Health Organization, 2018).

#### 2.4.1 Ligation/Bioconjugation Techniques Based on Multicomponent Reactions

Multicomponent reactions were also a powerful tool to generate covalent bonding, improving the conjugation between a broad range of biomolecules and cargos.

#### 2.4.2 Lipid-Lipid and Lipid-Oligosaccharide Conjugation

**FIGURE 9:**
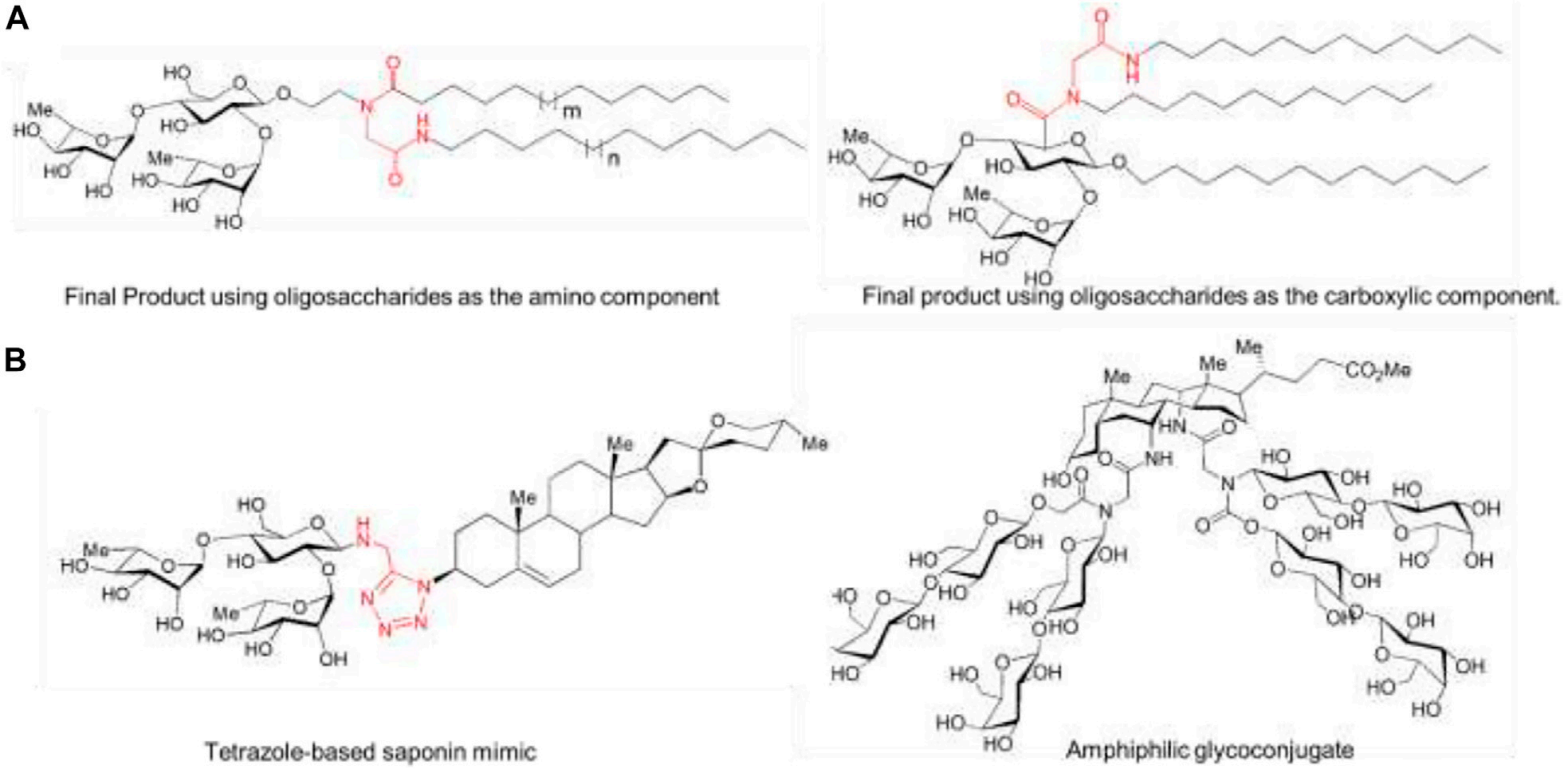
**(A)** Lipid-Lipid and lipid-oligosaccharide conjugates. **(B)** Oligosaccharide-steroid conjugates: Steroid-carbohydrate conjugates by multicomponent conjugation (MCC) of oligosaccharide to steroidal isocyanides. The most important covalent bond between entities is highlighted in red.

A procedure based on MCR allows to conjugate two lipid tails to an oligosaccharide moiety by Ugi multicomponent reaction (U-MCR). Two variants were achieved in which a carbohydrate (oligosaccharides) is used as either the carboxylic acid or amino component. The oligosaccharides were functionalized with fatty acid and lipidic isocyanides, yielding glycolipids. Thus, this approach can multi-functionalize biomolecules in one spot synthesis, opening possibilities for accessing DDS analogs of anti-cancer glycolipids (Figure 9A) (Pérez-Labrada et al., 2012; Liu et al., 2019).

#### 2.4.3 Oligosaccharide-Steroid Conjugation

The synthesis of steroid-carbohydrate derivatives by I-MCRs was carried out with spirostanic steroids and β-chacotrioside functionalized as either the amino or carboxylic acid component producing a library of cytotoxic analogs of diosgenyl-β-chacotrioside (tetrazole-based saponin mimic) for anti-cancer activity (Rivera et al., 2012) (Figure 9B). Even, to obtain more complex conjugation, it is ligated two oligosaccharide moieties to a bifunctional steroid, conjugation of 4 lactosyl fragments and acholic acid-derived di-isocyanide by means of a double Ugi reaction, rendering steroidal glycoconjugate (amphiphilic glycoconjugate), which the lactose units are located at the α face of concave cholanic skeleton (Rivera et al., 2013) (Figure 9B).

#### 2.4.4 Peptide-Peptide and Carbohydrate-Peptide Ligation

**FIGURE 10:**
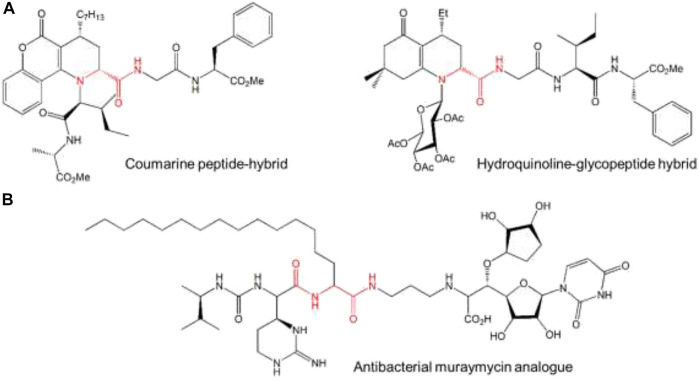
Peptide-peptide and carbohydrate peptide conjugation. **(A)** The final product of diastereoselective multicomponent ligation (MCL) of peptide and sugar residues to chiral bifunctional building blocks, **(B)** final product of MLC of urea-peptide, a lipid, and aminoribosyl-5-C-glycyludrine. The most important covalent bond between entities is highlighted in red.

The ligation of oligopeptides fragments by I-MCRs can be used as an approach for the Ugi multicomponent ligation (UML) of two pseudo peptides fragments to yield more stable and highly active peptides analogs using a highly diastereoselective UML procedure (Arabanian et al., 2009; Znabet et al., 2010; Pando et al., 2011). Moreover, the Ugi peptide ligation can be expanded by the development of the highly stereoselective organocatalytic MCR method for peptide-peptide or sugar-peptide conjugation, which is based on the use of enantiomerically enriched enol-hemiacetals as chiral inputs to conjugate isocyanopeptides to carbohydrates and amino peptides to form peptidic hybrids (Echemendía et al., 2015) (Figure 10A). Furthermore, the MCL of urea-peptide, a lipidic aldehyde, and a hybrid ribosyl-uridine isocyanide can produce a potent lipophilic antibacterial muraymicin analog (Figure 10B) (Tanino et al., 2010).

#### 2.4.5 Peptide-Steroids and Peptide-Lipid Conjugation

**FIGURE 11:**
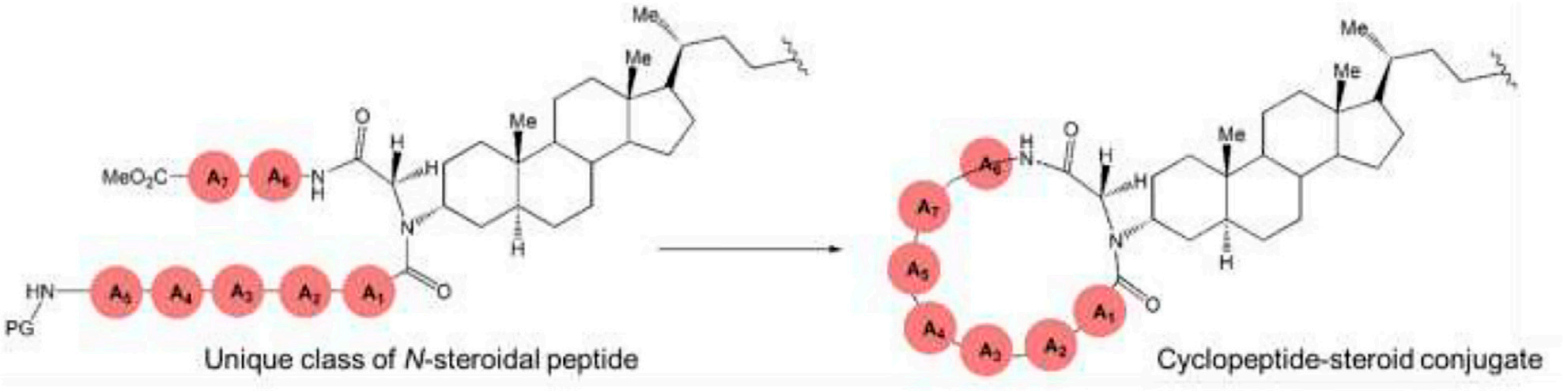
Peptide-steroid conjugation. Multicomponent conjugation (MCC) of two peptide fragments previously linked to the steroid to construct a unique *N*-steroidal cyclopeptide.

The synthesis of peptide-steroid conjugates can be achieved through multicomponent conjugation (MCC) of individual amino acids (A.A.s) (Rivera et al., 2006; Singla and Salunke, 2020). This concept can be expanded to creating a first-in-class family of peptide steroid conjugates (Unique class of *N*-steroidal peptide), highlighting the peptide backbone with the steroid skeleton as an N-substituent through an MCC of peptide carboxylic acid and isocyanopeptide or isocyanoacetate to a steroidal amine. These *N*-steroidal peptides can be further cyclized to yield cyclopeptide-steroid conjugate, in which the cyclopeptide residue can be found in different positions of either steroidal or the side chain (Figure 11) (Rivera et al., 2006; Singla and Salunke, 2020).

**FIGURE 12:**
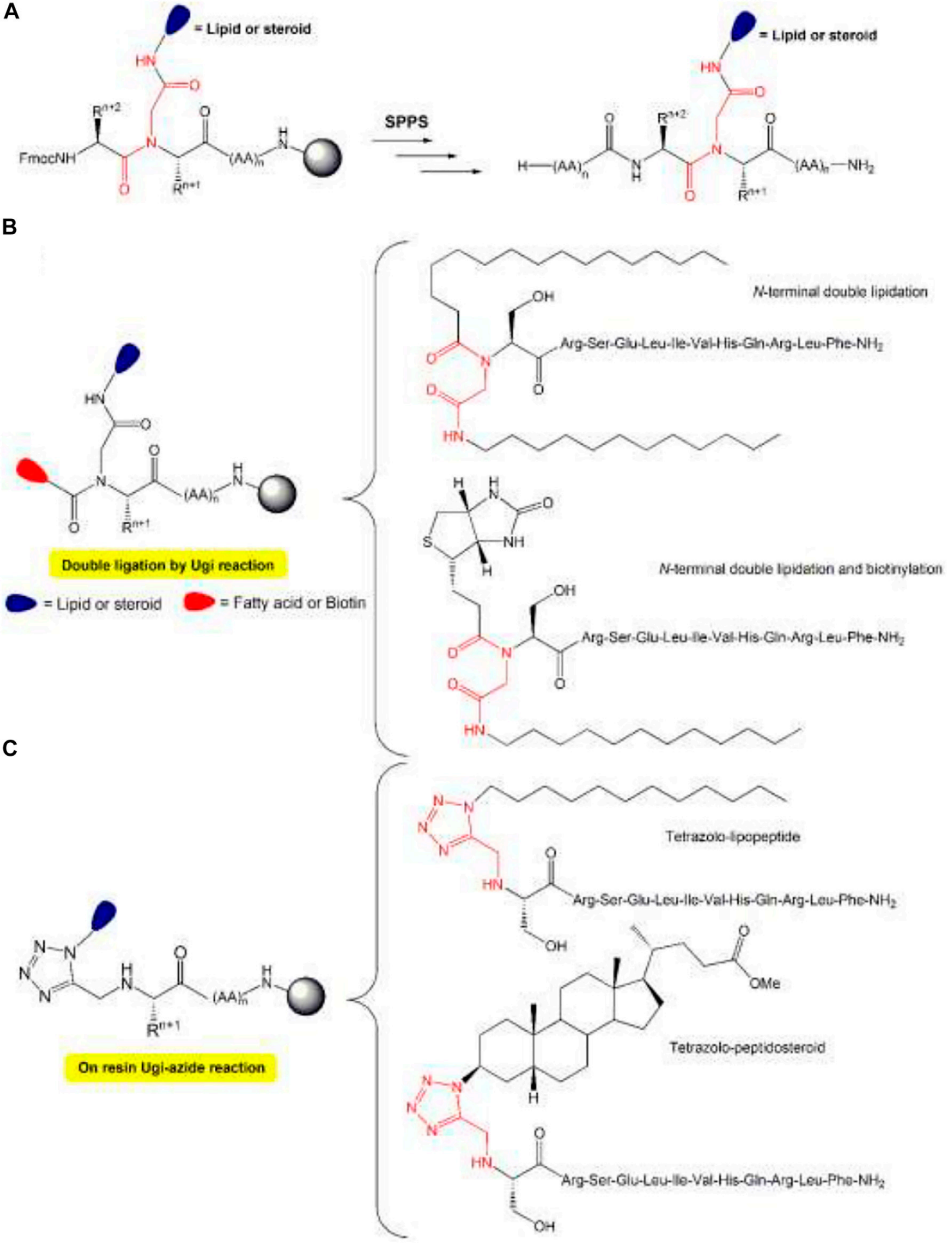
Peptide-steroid and peptide-lipid conjugates. **(A)** Solid phase ligation (SPL) of lipids and steroids by resin-bound peptide (RBP) using on-resin Ugi reaction, **(B)** double ligation by Ugi reaction, **(C)** SPL of lipids, biotinylated steroids by RBP using on-resin Ugi azide reaction. The most important covalent bond between entities is highlighted in red.

Moreover, the synthesis of antimicrobial lipopeptides can locate the lipidic tail either in the peptide termini as an internal amide or side-chain substituent (Morales et al., 2015; Wessjohann et al., 2016). The lipopeptides were created using a novel on-resin MCC of lipids and steroids to peptides based on solid-phase methodology, which enables the conjugation of either a lipid chain or a steroidal skeleton to the resin-bound peptide (RBP) (Figure 12A). Whereas subsequent A.A.s couplings allow the growth of the peptide, obtaining the desired lipopeptides and peptide-steroid conjugate. Similarly, a double ligation of an RBP at the *N*-terminus was carried out, allowing the *N*-terminal double lipidation and *N*-terminal lipidation and biotinylation (Figure 12B). Also, through a Ugi-azide reaction, it is obtained tetrazole lipopeptides and tetrazole peptidosteroid (Figure 12C) (Morales et al., 2015).

**FIGURE 13:**
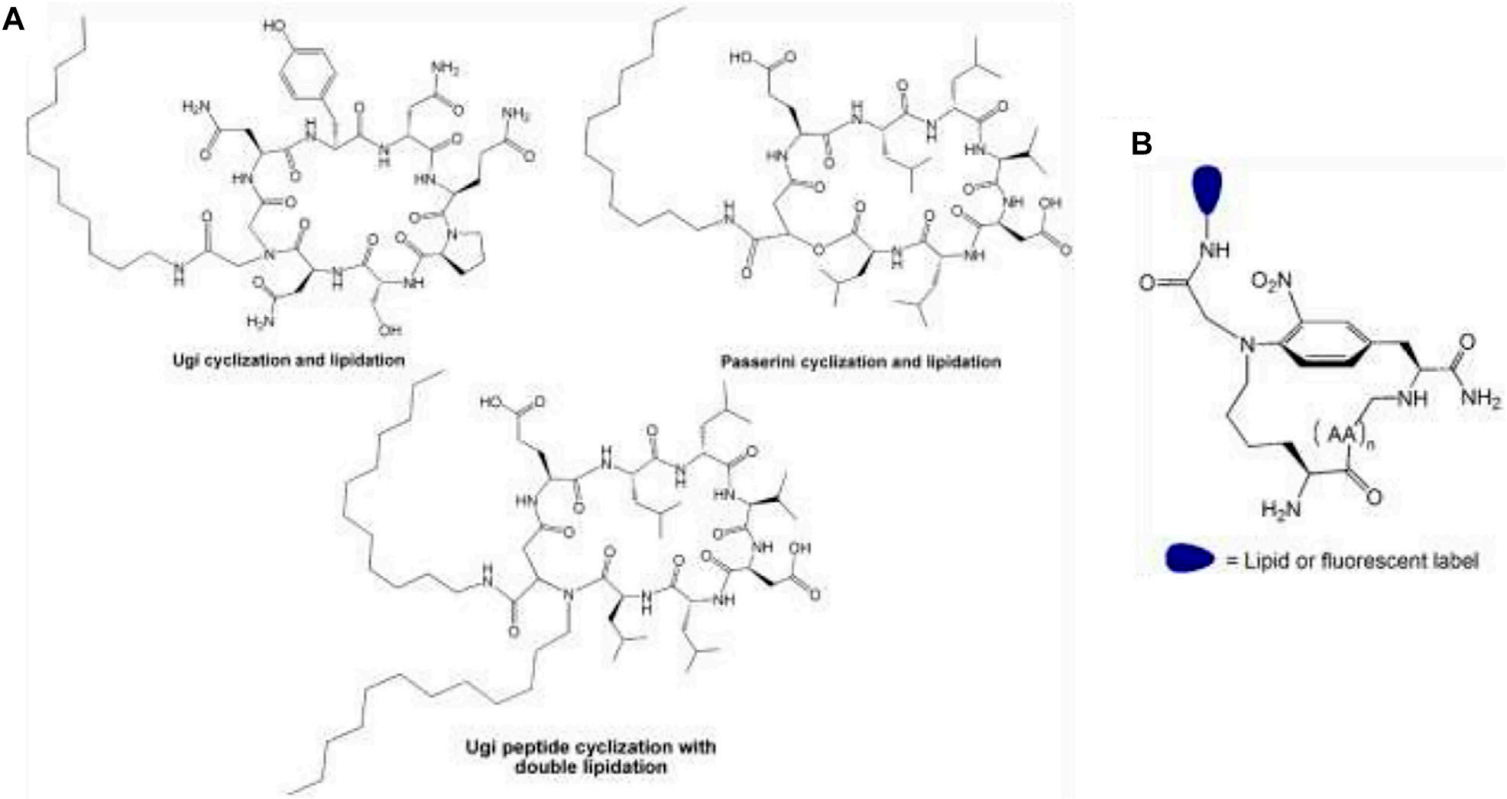
**(A)** Peptide-steroid and peptide-lipid conjugates. Products of simultaneous cyclization and lipidation of peptides by Ugi and Passerini reactions. **(B)** The pattern of the product was obtained using resin Ugi-smiles macrocyclization and ligation.

Furthermore, it can produce microbial natural products analogs (cyclic lipopeptides analogs) efficiently by Ugi and Passerini reactions to obtain a simultaneous cyclization and peptide lipidation. It let conjugate either one or two exocyclic lipid tails and the macrocyclic ring closure (Figure 13A) (Morejón et al., 2017). Likewise, the same procedure was proved by an on-resin Ugi-Smiles reaction creating a multicomponent cyclo-ligation strategy that enables the simultaneous cyclization of peptide skeleton and its ligation to either lipidic moieties or fluorescent labels (El Kaïm et al., 2005; Morejón et al., 2016) (Figure 13B).

Related to lipopeptides, a monocyte-targeting delivery platform based on cationic liposomes is based on functionalizing the liposomal membrane with a cholesterol-anchored tri-arginine peptide (TriArg) has been developed, guiding the design of future DDS utilized for immunotherapy (Münter et al., 2022). In the same way, a class of dendritic lipopeptide (DLP) liposomal delivery platform, consisting of a guanidinium-based dendritic peptide moiety, has been developed. The DDS is pH-sensitive and charge-reversible functions to enhance tumor accumulation and cell penetration (Münter et al., 2022).

#### 2.4.6 Protein Immobilization, Labeling, and Glycoconjugate

**FIGURE 14:**
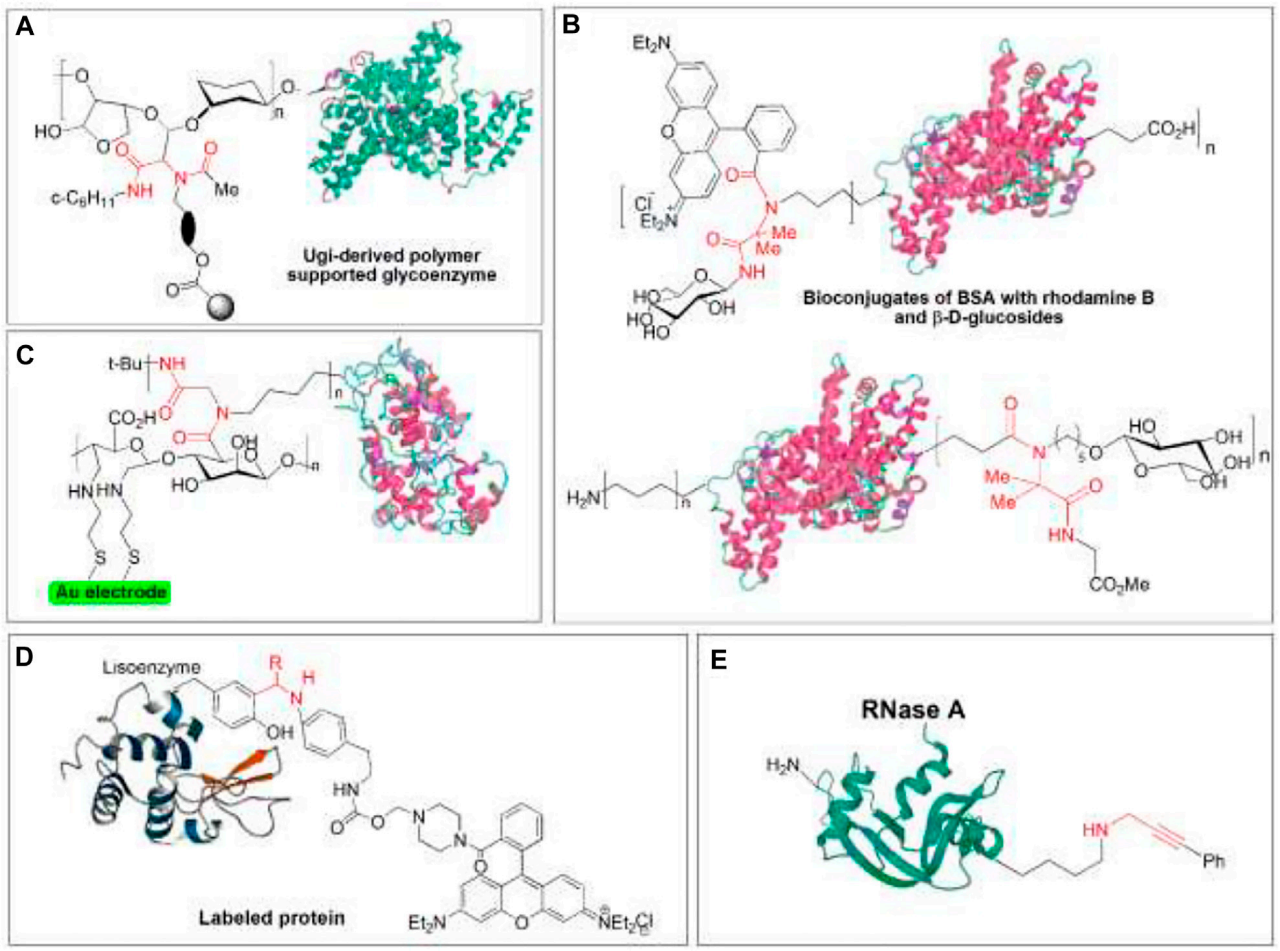
Protein immobilization, labeling, and glycoconjugate **(A)** Ugi-derived polymer-supported glycoenzyme; **(B)** final products from multicomponent conjugation (MCC) to polymeric support (see Panel **(A)**) and fluorescent label (rhodamine B) and carbohydrate (β-D-glucosides) using Ugi reaction; **(C)** multicomponent immobilization (MCI) of horseradish peroxidase (HRP) on a polysaccharide-coated gold electrode; **(D)** site-selective protein labeling at Tyr residue by Mannich type MCC; **(E)**. Site-selective protein labeling at only one Lys residue by Cu-catalyzed A3 coupling MCC. The most important covalent bond between entities is highlighted in red.

Protein immobilization can be achieved through efficient Ugi reaction procedures, immobilizing the enzyme glucose oxidase on a polymeric carrier. The glycoenzime undergoes periodate oxidation to obtain the aldehyde group in the glycosidic part. Then it is linked to “amino-functionalized glycidyl methacrylate polymer using an excess of acetic acid and cyclohexyl isocyanide” to produce the polymer-supported enzyme (Figure 14A) (Marek et al., 1983). Also, it was prepared bovine serum albumin (BSA) and horseradish peroxidase (HRP) conjugates by Ugi reaction using either the carboxylic acid or amino groups at the biomolecules surface, monosaccharides, and isocyano components to render several Ugi-derived glycoconjugates (Figure 14B). Although, this procedure has a problem due to the long reaction times that cause the protein denaturation as a result of protein crosslinking which can be solved by incorporating in Ugi reaction to increase protein reactivity of amino group and decrease reaction time (Ziegler et al., 2000; Méndez et al., 2018). Moreover, polysaccharide conjugation can be obtained by I-MCRs (Passerinin and Ugi reaction) to produce “biocompatible synthetic hydrogels” to immobilize enzymes in polysaccharide networks, which serve as carriers in DDS (De Nooy et al., 1999). In addition, an amperometric enzyme biosensor was developed for hydrogen peroxide immobilization of HRP on a gold electrode coated with sodium alginate by Ugi reaction (Figure 14C). This method consists of functionalizing polysaccharides with thiol groups by periodate oxidation and subsequent reductive amination. Once the polymer is attached to a gold electrode, it achieved electrode-immobilized enzyme using the Ugi bioconjugation procedure (Camacho et al., 2007). Similarly, the Ugi bioconjugation strategy is suitable for preparing thermostable neoglyconenzymes conjugating trypsin with sodium alginate and carboxymethyl cellulose yielding trypsin-polysaccharide glycoconjugates (García et al., 2009). In addition, protein labeling can be obtained through a Mannich-type multicomponent process conjugation at Tyr residues which consists of the mild reaction between the Tyr phenol ring and imines derived from aldehydes and electron-rich anilines carrying rhodamine tag to yield labeled proteins (Figure 14D) (Joshi et al., 2004) Another procedure of site-selective protein labeling based on MCRs is using Cu-catalyzed A^3^ couplings (Figure 14E) (Chilamari et al., 2017).

**FIGURE 15:**
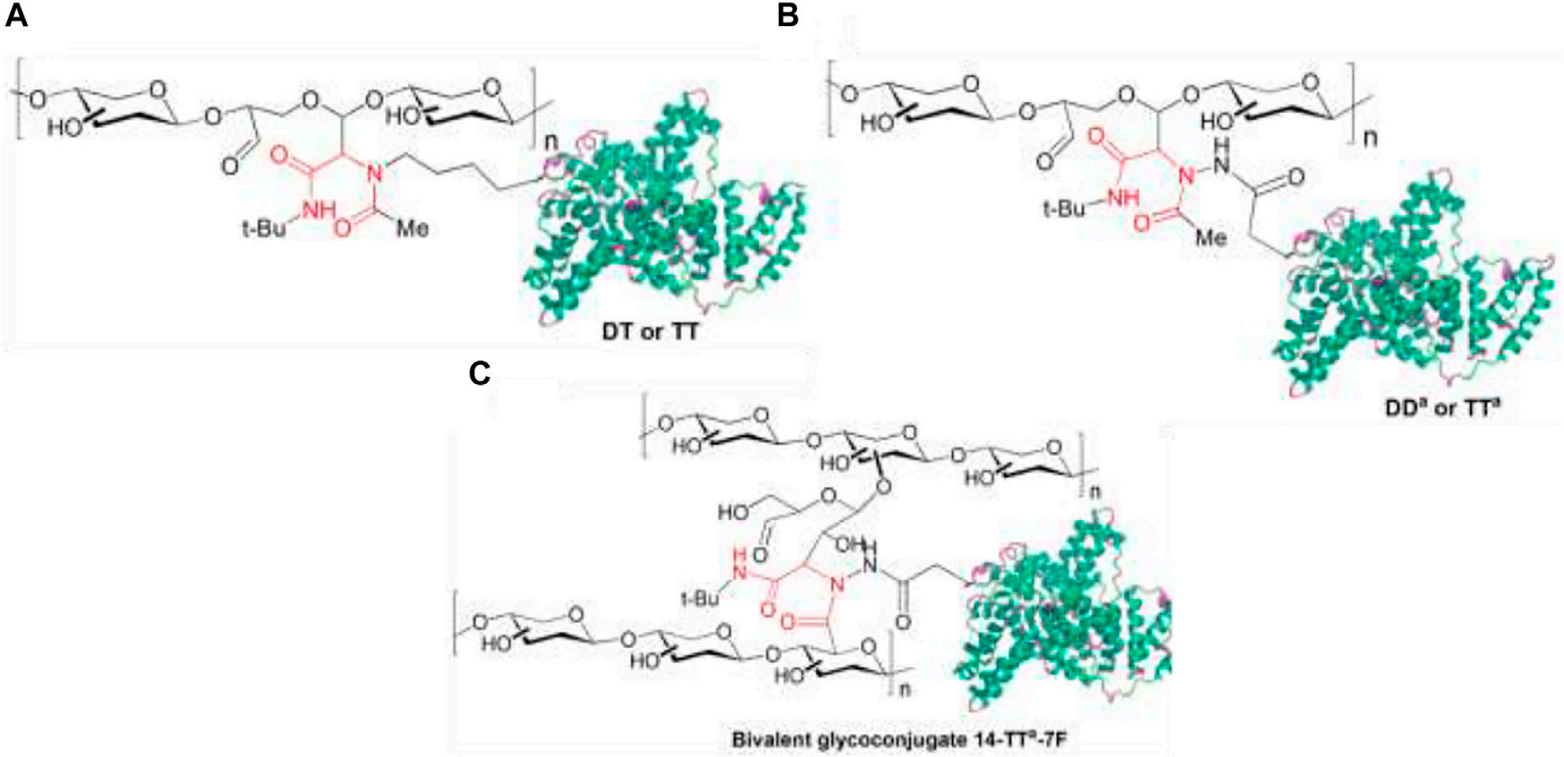
Final products obtained from protein immobilization, labeling, and glycoconjugate through **(A)** multicomponent conjugation (MCC) of oxo-functionalized capsular polysaccharide (CP) to nonactivated diphtheria (DT) and tetanus toxoids (TT); **(B)** hydrazide-activated DT and TT (DDa or TTa), and **(C)** MCC of two CPs to hydrazide-activated TT for the obtention of multivalent glycoconjugate vaccines. The most important covalent bond between entities is highlighted in red.

Finally, the development of the multicomponent protein-polysaccharide conjugation method, which consists of UML of functionalized capsular polysaccharide (CP) of *Streptococcus* and *Salmonella* to carrier proteins like diphtheria and tetanus toxoids (DT and TT), is activated by reaction of glutamic and aspartic acid side chains with hydrazine (DT^a^ and TT^a^). The products of this procedure showed significant antigenicity and elicited goods titer of functional specific antibodies (Figures 15A, B). Similarly, two different polysaccharides were conjugated to a protein using TEMPO-oxidized CPs14 (carboxylic component) and periodate oxidized CPs7F (oxo component) TT^a^ (amino component), yielding a glycoconjugate with two different polysaccharide antigens conjugated to a carrier protein, which has dual polysaccharide antigenicity. This proved that this multicomponent bioconjugation method is efficient for developing multivalent vaccine candidates (Méndez et al., 2018) (Figure 15C).

Polysaccharide-peptide and polysaccharide-protein conjugates are essential for targeted drug delivery into cells and tissue, and comprehensive reviews on its properties and applications as DDS have been published (Torres-Pérez et al., 2020; Walther and Zelikin, 2021; Kurbangalieva et al., 2022; Mohan et al., 2022).

## 3 Conclusion

DDS is an emergent field in Medicinal Chemistry. DDS based on Erythrocytes, Liposomes, Nanoparticles, and mainly mAb has been successfully developed, yielding entities able to avoid or minimize drug side effects or circumvent the resistance of the disease to certain drugs. Many of these conjugates are already on the market, and many others are already in clinical trials. Therefore, the search for more efficient DDS is continuously under development.

DDS has emerged as the potential solution to well-known drawbacks of drug administration, focusing on diseases like cancer and maximizing the effectiveness of the administered drugs by aiming them at the specific area affected by the disease. In this regard, erythrocytes, liposomes, nanoparticles, and antibodies have been covalently linked to a broad range of cargos, i.e., drugs, markers, and biomolecules, with promising bioapplications. Amide or disulfide formation was the chemical way most used to generate hybrid conjugates. On the other hand, multicomponent reactions (MCR) have been incorporated as a powerful tool in preparing conjugates, taking advantage of its proven efficiency and allowing the combination between lipids, oligosaccharides, steroids, and peptides. Despite the rapid development of DDS, nowadays, only a few DDSs based on liposomes nanoparticles or antibodies are successfully commercialized, which is expected considering the times associated with this kind of process (i.e., design, optimization, clinical trials, and final drug registration). The great variety of chemical reactions that can be adapted to different carriers gives this topic great importance that will be reflected in an increase in related research and the number of conjugates in the market and those that will be approved by the FDA shortly.

After much time, appreciable technological advances, a better understanding of controlled drug release systems, and resources invested in the research and development of these systems, there are tangible results. The approval of several ADCs focused on cancer therapy and extensive research focused on the discovery of novel systems that use erythrocytes, liposomes, nanoparticles, or proteins, indicated a promising future. We believe the true potential of using covalent conjugation applied in DDS as a pharmacological platform is only just being realized, understood, and exploited.

## References

[B1] Adams D. Gonzalez-Duarte A. O’Riordan W. D. Yang C.-C. Ueda M. Kristen A. V. (2018). Patisiran, an RNAi Therapeutic, for Hereditary Transthyretin Amyloidosis. N. Engl. J. Med. 379, 11–21. 10.1056/nejmoa1716153 29972753

[B2] Aguilar-Pérez K. M. Avilés-Castrillo J. I. Medina D. I. Parra-Saldivar R. Iqbal H. M. N. (2020). Insight into Nanoliposomes as Smart Nanocarriers for Greening the Twenty-First Century Biomedical Settings. Front. Bioeng. Biotechnol. 8, 1441. 10.3389/fbioe.2020.579536 PMC7770187 33384988

[B3] Akbarzadeh A. Rezaei-sadabady R. Davaran S. Joo S. W. Zarghami N. (2013). Liposome : Classification , Preparation , and Applications. Nanoscale Res. Lett. 1, 9. 10.1186/1556-276X-8-102 PMC3599573 23432972

[B4] Alapan Y. Yasa O. Schauer O. Giltinan J. Tabak A. F. Sourjik V. (2018). Soft Erythrocyte-Based Bacterial Microswimmers for Cargo Delivery. Sci. Robot. 3, eaar4423. 10.1126/scirobotics.aar4423 33141741

[B162] Alwattar J. K. Mneimneh A. T. Abla K. K. Mehanna M. M. Allam A. N. (2021). Smart Stimuli-Responsive Liposomal Nanohybrid Systems: A Critical Review of Theranostic Behavior in Cancer. Pharmaceutics 13, 355. 10.3390/pharmaceutics13030355 33800292 PMC7999181

[B5] Amin Y. (2008). Highlights of Prescribing Information. Of, H., Of, H., Information, P., and Information, P. White Oak, Maryland: Lulu.com. Available at: www.fda.gov/medwatch (Accessed February 24, 2022).

[B6] Andriyanov A. V. Koren E. Barenholz Y. Goldberg S. N. (2014). Therapeutic Efficacy of Combining PEGylated Liposomal Doxorubicin and Radiofrequency (RF) Ablation: Comparison between Slow-Drug-Releasing, Non-thermosensitive and Fast-Drug-Releasing, Thermosensitive Nano-Liposomes. PLoS One 9. 10.1371/journal.pone.0092555 PMC4006748 24786533

[B7] Anselmo A. C. Mitragotri S. (2019). Nanoparticles in the Clinic: An Update. Bioeng. Transl. Med. 4. 10.1002/btm2.10143 PMC6764803 31572799

[B8] Arabanian A. Mohammadnejad M. Balalaie S. Gross J. H. (2009). Synthesis of Novel Gn-RH Analogues Using Ugi-4MCR. Bioorg. Med. Chem. Lett. 10.1016/j.bmcl.2008.11.111 19103486

[B9] Arcella A. Oliva M. A. Staffieri S. Aalberti S. Grillea G. Madonna M. (2015). *In Vitro* and *In Vivo* Effect of Human Lactoferrin on Glioblastoma Growth. J. Neurosurg. 123, 1026–1035. 10.3171/2014.12.JNS14512 26186026

[B10] Bang K. Cheon J. Jeong J. H. Im H. S. Kim K. P. Ryoo B. Y. (2021). Clinical Outcomes of Liposomal Irinotecan Plus Fluorouracil/leucovorin for Metastatic Pancreatic Adenocarcinoma in Patients Previously Treated with Conventional Irinotecan-Containing Chemotherapy. Ther. Adv. Med. Oncol. 13. 10.1177/17588359211003053 PMC7983461 33796153

[B49] Baron J. M. Boster B. L. Barnett C. M. (2015). Ado-Trastuzumab Emtansine (T-DM1): A Novel Antibody-Drug Conjugate for the Treatment of HER2-Positive Metastatic Breast Cancer J. Oncol. Pharm. Pract. 21, 132–142. 10.1177/1078155214527144 24682654

[B11] Bonvalot S. Le Pechoux C. De Baere T. Kantor G. Buy X. Stoeckle E. (2017). First-in-human Study Testing a New Radioenhancer Using Nanoparticles (NBTXR3) Activated by Radiation Therapy in Patients with Locally Advanced Soft Tissue Sarcomas. Clin. Cancer Res. 23, 908–917. 10.1158/1078-0432.CCR-16-1297 27998887

[B12] Bonvalot S. Rutkowski P. L. Thariat J. Carrère S. Ducassou A. Sunyach M. P. (2019). NBTXR3, a First-In-Class Radioenhancer Hafnium Oxide Nanoparticle, Plus Radiotherapy versus Radiotherapy Alone in Patients with Locally Advanced Soft-Tissue Sarcoma (Act.In.Sarc): a Multicentre, Phase 2–3, Randomised, Controlled Trial. Lancet Oncol. 20, 1148–1159. 10.1016/S1470-2045(19)30326-2 31296491

[B13] Borek A. Sokolowska-Wedzina A. Chodaczek G. Otlewski J. (2018). Generation of High-Affinity, Internalizing Anti-fgfr2 Single-Chain Variable Antibody Fragment Fused with Fc for Targeting Gastrointestinal Cancers. PLoS One 13, 1–16. 10.1371/journal.pone.0192194 PMC5805272 29420662

[B14] Bourgeaux V. Lanao J. Bax B. Yann G. (2016). Drug-loaded Erythrocytes: On the Road toward Marketing Approval. Drug Des. Devel. Ther. 10, 665–676. 10.2147/DDDT.S96470.LK PMC4755692 26929599

[B15] Brentuximab Vedotin (SGN35) (2013). Drug Description. J. Antib. Drug Conjug. Available at: https://www.adcreview.com/brentuximab-vedotin-sgn35/.

[B16] Briolay T. Petithomme T. Fouet M. Nguyen-Pham N. Blanquart C. Boisgerault N. (2021). Delivery of Cancer Therapies by Synthetic and Bio-Inspired Nanovectors. Mol. Cancer, 1–24. 2021 201 20. 10.1186/S12943-021-01346-2 33761944 PMC7987750

[B17] Burande A. S. Viswanadh M. K. Jha A. Mehata A. K. Shaik A. Agrawal N. (2020). EGFR Targeted Paclitaxel and Piperine Co-loaded Liposomes for the Treatment of Triple Negative Breast Cancer. AAPS PharmSciTech 21, 1–12. 10.1208/s12249-020-01671-7 32440910

[B18] Caimi P. F. Ai W. Alderuccio J. P. Ardeshna K. M. Hamadani M. Hess B. (2021). Loncastuximab Tesirine in Relapsed or Refractory Diffuse Large B-Cell Lymphoma (LOTIS-2): a Multicentre, Open-Label, Single-Arm, Phase 2 Trial. Lancet Oncol. 22, 790–800. 10.1016/S1470-2045(21)00139-X 33989558

[B19] Camacho C. Matías J. C. García D. Simpsom B. K. Villalonga R. (2007). Amperometric Enzyme Biosensor for Hydrogen Peroxide via Ugi Multicomponent Reaction. Electrochem. Commun. 9, 1655–1660. 10.1016/j.elecom.2007.03.013

[B20] Cevaal P. M. Ali A. Czuba-Wojnilowicz E. Symons J. Lewin S. R. Cortez-Jugo C. (2021). *In Vivo* T Cell-Targeting Nanoparticle Drug Delivery Systems: Considerations for Rational Design. ACS Nano 15, 3736–3753. 10.1021/acsnano.0c09514 33600163

[B22] Chandan R. Banerjee R. (2018). Pro-apoptotic Liposomes-Nanobubble Conjugate Synergistic with Paclitaxel: A Platform for Ultrasound Responsive Image-Guided Drug Delivery. Sci. Rep. 8, 1–15. 10.1038/s41598-018-21084-8 29422676 PMC5805674

[B23] Chang T. C. Shiah H. S. Yang C. H. Yeh K. H. Cheng A. L. Shen B. N. (2015). Phase I Study of Nanoliposomal Irinotecan (PEP02) in Advanced Solid Tumor Patients. Cancer Chemother. Pharmacol. 75, 579–586. 10.1007/s00280-014-2671-x 25577133 PMC4341010

[B24] Chilamari M. Purushottam L. Rai V. (2017). Site-Selective Labeling of Native Proteins by a Multicomponent Approach. Chem. - A. Eur. J. 10.1002/chem.201605938 28177162

[B25] Chiu D. Pan L. Fay L. Eakin C. Valliere-Douglass J. (2021). Structural Characterization of a Monomethylauristatin-E Based ADC that Contains 8 Drugs Conjugated at Interchain Cysteine Residues. J. Pharm. Biomed. Anal. 205, 114309. 10.1016/j.jpba.2021.114309 34403866

[B26] Coleman R. L. Lorusso D. Gennigens C. González-Martín A. Randall L. Cibula D. (2021). Efficacy and Safety of Tisotumab Vedotin in Previously Treated Recurrent or Metastatic Cervical Cancer (innovaTV 204/GOG-3023/engot-Cx6): a Multicentre, Open-Label, Single-Arm, Phase 2 Study. Lancet Oncol. 22, 609–619. 10.1016/S1470-2045(21)00056-5 33845034

[B27] Craciunescu O. Icriverzi M. Florian P. E. Roseanu A. Trif M. (2021). Mechanisms and Pharmaceutical Action of Lipid Nanoformulation of Natural Bioactive Compounds as Efficient Delivery Systems in the Therapy of Osteoarthritis. Pharmaceutics 13, 1108. 10.3390/pharmaceutics13081108 34452068 PMC8399940

[B28] Dag A. Jiang Y. Karim K. J. A. Hart-Smith G. Scarano W. Stenzel M. H. (2015). Polymer-albumin Conjugate for the Facilitated Delivery of Macromolecular Platinum Drugs. Macromol. Rapid Commun. 36, 890–897. 10.1002/marc.201400576 25790077

[B29] Daghistani N. Rey J. A. (2016). Invega Trinza: The First Four-Times-A-Year, Long-Acting Injectable Antipsychotic Agent. Pharm. Ther. 41, 222. PMC4811251 27069340

[B30] De Goeij B. E. C. G. Satijn D. Freitag C. M. Wubbolts R. Bleeker W. K. Khasanov A. (2015). High Turnover of Tissue Factor Enables Efficient Intracellular Delivery of Antibody-Drug Conjugates. Mol. Cancer Ther. 14, 1130–1140. 10.1158/1535-7163.MCT-14-0798 25724665

[B31] De Matteis V. Rinaldi R. (2018). “Toxicity Assessment in the Nanoparticle Era,” in Advances in Experimental Medicine and Biology (Cham: Springer), 1–19. 10.1007/978-3-319-72041-8_1 29453529

[B32] De Nooy A. E. J. Masci G. Crescenzi V. (1999). Versatile Synthesis of Polysaccharide Hydrogels Using the Passerini and Ugi Multicomponent Condensations. Macromolecules 32 (4), 1318–1320. 10.1021/ma9815455

[B33] Dhritlahre R. K. Saneja A. (2021). Recent Advances in HER2-Targeted Delivery for Cancer Therapy. Drug DiscovToday 26, 1319–1329. 10.1016/j.drudis.2020.12.014 33359114

[B34] Diamantis N. Banerji U. (2016). Antibody-drug Conjugates - an Emerging Class of Cancer Treatment. Br. J. Cancer 114, 362–367. 10.1038/bjc.2015.435 26742008 PMC4815767

[B35] do Pazo C. Nawaz K. Webster R. M. (2021). The Oncology Market for Antibody-Drug Conjugates. Nat. Rev. Drug Discov. 20, 583–584. 10.1038/d41573-021-00054-2 33762691

[B36] Doronina S. O. Toki B. E. Torgov M. Y. Mendelsohn B. A. Cerveny C. G. Chace D. F. (2003). Development of Potent Monoclonal Antibody Auristatin Conjugates for Cancer Therapy. Nat. Biotechnol. 21, 778–784. 10.1038/nbt832 12778055

[B37] Echemendía R. De La Torre A. F. Monteiro J. L. Pila M. Corrêa A. G. Westermann B. (2015). Highly Stereoselective Synthesis of Natural-product-like Hybrids by an Organocatalytic/multicomponent Reaction Sequence. Angew. Chem. Int. Ed. 54 (26), 7621–7625. 10.1002/anie.201412074 25967546

[B38] El Kaïm L. Grimaud L. Oble J. (2005). Phenol Ugi-Smiles Systems: Strategies for the Multicomponent N-Arylation of Primary Amines with Isocyanides, Aldehydes, and Phenols. Angew. Chem. - Int. Ed. 44 (48), 7961–7964. 10.1002/anie.200502636 16287162

[B116] European Medicines Agency (2013). Krystexxa European Medicines Agency. Available at: https://www.ema.europa.eu/en/medicines/human/EPAR/krystexxa (Accessed May 2, 2022).

[B39] Ferraro E. Drago J. Z. Modi S. (2021). Implementing Antibody-Drug Conjugates (ADCs) in HER2-Positive Breast Cancer: State of the Art and Future Directions. Breast Cancer Res. 23, 1–11. 10.1186/s13058-021-01459-y 34380530 PMC8356386

[B40] Friedman A. Claypool S. Liu R. (2013). The Smart Targeting of Nanoparticles. Curr. Pharm. Des. 19, 6315–6329. 10.2174/13816128113199990375 23470005 PMC4016770

[B41] Gabizon A. A. (2001). Pegylated Liposomal Doxorubicin: Metamorphosis of an Old Drug into a New Form of Chemotherapy. Cancer Invest. 19, 424–436. 10.1081/cnv-100103136 11405181

[B42] Ganguly K. Krasik T. Medinilla S. Bdeir K. Cines D. B. Muzykantov V. R. (2005). Blood Clearance and Activity of Erythrocyte-Coupled Fibrinolytics. J. Pharmacol. Exp. Ther. 312, 1106–1113. 10.1124/jpet.104.075770.Wooster 15525799

[B43] Ganguly K. Murciano J. Westrick R. Leferovich J. Cines D. B. Muzykantov V. R. (2007). The Glycocalyx Protects Erythrocyte-Bound Tissue-type Plasminogen Activator from Enzymatic Inhibition. J. Pharmacol. Exp. Ther. 321, 158–164. 10.1124/jpet.106.114405.plasma 17215448

[B44] García A. Hernández K. Chico B. García D. Villalonga M. L. Villalonga R. (2009). Preparation of Thermostable Trypsin-Polysaccharide Neoglycoenzymes through Ugi Multicomponent Reaction. J. Mol. Catal. B Enzym. 10.1016/j.molcatb.2009.02.001

[B45] Glassman P. M. Villa C. H. Ukidve A. Zhao Z. Smith P. Mitragotri S. (20202020). Vascular Drug Delivery Using Carrier Red Blood Cells: Focus on RBC Surface Loading and Pharmacokinetics. Pharm 12 (440 12), 440. 10.3390/PHARMACEUTICS12050440 PMC7284780 32397513

[B46] Goss G. D. Vokes E. E. Gordon M. S. Gandhi L. Papadopoulos K. P. Rasco D. W. (2018). Efficacy and Safety Results of Depatuxizumab Mafodotin (ABT-414) in Patients with Advanced Solid Tumors Likely to Overexpress Epidermal Growth Factor Receptor. Cancer 124, 2174–2183. 10.1002/cncr.31304 29533458 PMC5969257

[B47] Grimm A. J. Kontos S. Diaceri G. Quaglia-Thermes X. Hubbell J. A. (2015b). Memory of Tolerance and Induction of Regulatory T Cells by Erythrocyte-Targeted Antigens. Sci. Rep. 5, 1–11. 10.1038/srep15907 PMC4625129 26511151

[B48] Grimm A. J. Stephan Kontos Giacomo Diaceri X. Q.-T. Hubbell J. A. (2015a). Memory of Tolerance and Induction of Regulatory T Cells by Erythrocyte-Targeted Antigens. Sci. Rep. 5, 1–11. 10.1038/srep15907 PMC4625129 26511151

[B50] Halfon-Domenech C. Thomas X. Chabaud S. Baruchel A. Gueyffier F. Mazingue F. (2011). l-Asparaginase Loaded Red Blood Cells in Refractory or Relapsing Acute Lymphoblastic Leukaemia in Children and Adults: Results of the GRASPALL 2005-01 Randomized Trial. Br. J. Haematol. 153, 58–65. 10.1111/j.1365-2141.2011.08588.x 21332712

[B51] Hamann P. R. Hinman L. M. Hollander I. Beyer C. F. Lindh D. Holcomb R. (2002). Gemtuzumab Ozogamicin, a Potent and Selective Anti-CD33 Antibody - Calicheamicin Conjugate for Treatment of Acute Myeloid Leukemia. Bioconjug. Chem. 13, 47–58. 10.1021/bc010021y 11792178

[B52] Hammel P. Fabienne P. Mineur L. Metges J. P. Andre T. De La Fouchardiere C. (2020). Erythrocyte-encapsulated Asparaginase (Eryaspase) Combined with Chemotherapy in Second-Line Treatment of Advanced Pancreatic Cancer: An Open-Label, Randomized Phase IIb Trial. Eur. J. Cancer 124, 91–101. 10.1016/j.ejca.2019.10.020 31760314

[B53] Huang F. Y. J. Chen W. J. Lee W. Y. Lo S. T. Lee T. W. Lo J. M. (2013). *In Vitro* and *In Vivo* Evaluation of Lactoferrin-Conjugated Liposomes as a Novel Carrier to Improve the Brain Delivery. Int. J. Mol. Sci. 14, 2862–2874. 10.3390/ijms14022862 23434652 PMC3588019

[B54] Irby D. Du C. Li F. (2017). Lipid-Drug Conjugate for Enhancing Drug Delivery. Mol. Pharm. 14, 1325–1338. 10.1021/acs.molpharmaceut.6b01027 28080053 PMC5477224

[B55] Jacob J. Haponiuk J. T. Thomas S. Gopi S. (2018). Biopolymer Based Nanomaterials in Drug Delivery Systems: A Review. Mater. Today Chem. 9, 43–55. 10.1016/j.mtchem.2018.05.002

[B56] Javed S. Alshehri S. Shoaib A. Ahsan W. Sultan M. H. Alqahtani S. S. (20212021). Chronicles of Nanoerythrosomes: An Erythrocyte-Based Biomimetic Smart Drug Delivery System as a Therapeutic and Diagnostic Tool in Cancer Therapy. Pharm 13, 368. 368 13. 10.3390/PHARMACEUTICS13030368 PMC7998655 33802156

[B57] Jeong W. Y. Kwon M. Choi H. E. Kim K. S. (2021a). Recent Advances in Transdermal Drug Delivery Systems: a Review. Biomater. Res. 25, 1–15. 10.1186/s40824-021-00226-6 34321111 PMC8317283

[B58] Jeong W. Y. Kwon M. Choi H. E. Kim K. S. (2021b2021). Recent Advances in Transdermal Drug Delivery Systems: a Review. Biomater. Res. 251 25, 1–15. 10.1186/S40824-021-00226-6 PMC8317283 34321111

[B59] Jiang Y. Liang M. Svejkar D. Hart-Smith G. Lu H. Scarano W. (2014). Albumin-micelles via a One-Pot Technology Platform for the Delivery of Drugs. Chem. Commun. 50, 6394–6397. 10.1039/c4cc00616j 24811583

[B60] Joshi N. S. Whitaker L. R. Francis M. B. (2004). A Three-Component Mannich-type Reaction for Selective Tyrosine Bioconjugation. J. Am. Chem. Soc. 126, 15942–15943. 10.1021/ja0439017 15584710

[B61] Kanojia N. Sharma N. Gupta N. Singh S. (2022). Applications of Nanostructured Lipid Carriers: Recent Advancements and Patent Review. Biointerface Res. Appl. Chem. 12, 638–652. 10.33263/BRIAC121.638652

[B62] Khattak Z. E. Hashmi H. Khan S. I. Aamir S. Arif U. Khan A. I. (2021). Dawn of a new era of Antibody-Drug Conjugates and Bispecific T-Cell Engagers for Treatment of Multiple Myeloma: a Systematic Review of Literature. Ann. Hematol. 100, 2155–2172. 10.1007/s00277-021-04599-5 34318356

[B63] Köhler G. Milstein C. (19921975). Continuous Cultures of Fused Cells Secreting Antibody of Predefined Specificity. Biotechnology 24, 524–526. 10.1038/256495a0 1422065

[B64] Kostova V. Désos P. Starck J. B. Kotschy A. (2021). The Chemistry behind Adcs. Pharmaceuticals 14, 442. 10.3390/ph14050442 34067144 PMC8152005

[B65] Krauss A. C. Gao X. Li L. Manning M. L. Patel P. Fu W. (2019). FDA Approval Summary: (Daunorubicin and Cytarabine) Liposome for Injection for the Treatment of Adults with High-Risk Acute Myeloid Leukemia. Clin. Cancer Res. 25, 2685–2690. 10.1158/1078-0432.CCR-18-2990 30541745

[B66] Kumar A. Kinneer K. Masterson L. Ezeadi E. Howard P. Wu H. (2018). Synthesis of a Heterotrifunctional Linker for the Site-specific Preparation of Antibody-Drug Conjugates with Two Distinct Warheads. Bioorg. Med. Chem. Lett. 28, 3617–3621. 10.1016/j.bmcl.2018.10.043 30389292

[B67] Kurbangalieva A. Zamalieva R. Nasibullin I. Yamada K. Tanaka K. (2022). Homo- and Heterogeneous Glycoconjugates on the Basis of N-Glycans and Human Serum Albumin: Synthesis and Biological Evaluation. Mol 202227, 1285. *Page 1285* 27. 10.3390/MOLECULES27041285 PMC8877828 35209074

[B68] Lanigan R. M. Karaluka V. Sabatini M. T. Starkov P. Badland M. Boulton L. (2016). Direct Amidation of Unprotected Amino Acids Using B(OCH _2_ CF _3_ ) _3_ . Chem. Commun. 52, 8846–8849. 10.1039/C6CC05147B 27346362

[B69] Lejeune A. Moorjani M. Gicquaud C. Lacroix J. Poyet P. Gaudreault C. R. (1994). Nanoerythrosome, a New Derivative of Erythrocyte Ghost: Preparation and Antineoplastic Potential as Drug Carrier for Daunorubicin. Anticancer Res. 14, 915–919. Available at: https://pubmed.ncbi.nlm.nih.gov/8074493/. 8074493

[B70] Levengood M. R. Zhang X. Hunter J. H. Emmerton K. K. Miyamoto J. B. Lewis T. S. (2017). Orthogonal Cysteine Protection Enables Homogeneous Multi-Drug Antibody–Drug Conjugates. Angew. Chem. Int. Ed. 56, 733–737. 10.1002/ANIE.201608292 PMC5299463 27966822

[B71] Liao C. C. Yu H. P. Yang S. C. Alalaiwe A. Dai Y. S. Liu F. C. (2021). Multifunctional Lipid-Based Nanocarriers with Antibacterial and Anti‐inflammatory Activities for Treating MRSA Bacteremia in Mice. J. Nanobiotechnology 19, 1–18. 10.1186/s12951-021-00789-5 33588861 PMC7885212

[B72] Lin C. Wong B. C. K. Chen H. Bian Z. Zhang G. Zhang X. (2017). Pulmonary Delivery of Triptolide-Loaded Liposomes Decorated with Anti-carbonic Anhydrase IX Antibody for Lung Cancer Therapy. Sci. Rep. 7, 1–12. 10.1038/s41598-017-00957-4 28428618 PMC5430522

[B73] Liu Q. Li X. Bao Y. S. Lu J. Li H. Huang Z. (2019). Chemical Synthesis and Functional Characterization of a New Class of Ceramide Analogues as Anti-cancer Agents. Bioorg. Med. Chem. 27, 1489–1496. 10.1016/j.bmc.2019.02.030 30837168

[B74] Liu Y. Zhang B. Xu J. Wang X. Tang J. Huang J. (2021). Phase I Study of Liposomal Irinotecan (LY01610) in Patients with Advanced Esophageal Squamous Cell Carcinoma. Cancer Chemother. Pharmacol. 88, 403–414. 10.1007/s00280-021-04294-2 34031756 PMC8143070

[B75] Lorentz K. M. Kontos S. Diaceri G. Henry H. Hubbell J. A. (2015). Engineered Binding to Erythrocytes Induces Immunological Tolerance to *E. coli* Asparaginase. Sci. Adv. 1, 1500112. 10.1126/sciadv.1500112 PMC4646778 26601215

[B76] Lu J. Jiang F. Lu A. Zhang G. (2016). Linkers Having a Crucial Role in Antibody–Drug Conjugates. Int. J. Mol. Sci. 17, 561. 10.3390/ijms17040561 27089329 PMC4849017

[B77] Lungu M. Neculae A. Bunoiu M. Biris C. (2015). Nanoparticles’ Promises and Risks: Characterization, Manipulation, and Potential Hazards to Humanity and the Environment. Nanoparticles’ Promises Risks Charact. Manip. Potential Hazards Humanit. Environ., 1–355. 10.1007/978-3-319-11728-7

[B78] Luo G.-F. Chen W.-H. Zeng X. Zhang X.-Z. (2021). Cell Primitive-Based Biomimetic Functional Materials for Enhanced Cancer Therapy. Chem. Soc. Rev. 50, 945–985. 10.1039/D0CS00152J 33226037

[B79] Marek M. Jarý J. Valentová O. Vodrážka Z. (1983). Immobilization of Glycoenzymes by Means of Their Glycosidic Components. Biotechnol. Lett. 5, 653–658. 10.1007/BF01386357

[B80] McCombs J. R. Owen S. C. (2015). Antibody Drug Conjugates: Design and Selection of Linker, Payload and Conjugation Chemistry. AAPS J. 17, 339–351. 10.1208/s12248-014-9710-8 25604608 PMC4365093

[B82] Méndez Y. Chang J. Humpierre A. R. Zanuy A. Garrido R. Vasco A. V. (2018). Multicomponent Polysaccharide-Protein Bioconjugation in the Development of Antibacterial Glycoconjugate Vaccine Candidates. Chem. Sci. 9 (9), 2581–2588. 10.1039/c7sc05467j 29719713 PMC5897956

[B83] Milton Harris J. Martin N. E. Modi M. (2001). Pegylation: A Novel Process for Modifying Pharmacokinetics. Clin. Pharmacokinet. 40, 539–551. 10.2165/00003088-200140070-00005 11510630

[B84] Mohan T. Kleinschek K. S. Kargl R. (2022). Polysaccharide Peptide Conjugates: Chemistry, Properties and Applications. CarbohydrPolym 280, 118875. 10.1016/J.CARBPOL.2021.118875 35027118

[B85] Morales F. E. Garay H. E. Muñoz D. F. Augusto Y. E. Otero-González A. J. Reyes Acosta O. (2015). Aminocatalysis-mediated On-Resin Ugi Reactions: Application in the Solid-phase Synthesis of N -substituted and Tetrazolo Lipopeptides and Peptidosteroids. Org. Lett. 10.1021/acs.orglett.5b01147 25994574

[B86] Morejón M. C. Laub A. Kaluderović G. N. Puentes A. R. Hmedat A. N. Otero-González A. J. (2017). A Multicomponent Macrocyclization Strategy to Natural Product-like Cyclic Lipopeptides: Synthesis and Anticancer Evaluation of Surfactin and Mycosubtilin Analogues. Org. Biomol. Chem. 15 (17), 3628–3637. 10.1039/c7ob00459a 28406518

[B87] Morejón M. C. Laub A. Westermann B. Rivera D. G. Wessjohann L. A. (2016). Solution- and Solid-phase Macrocyclization of Peptides by the Ugi-Smiles Multicomponent Reaction: Synthesis of N-Aryl-Bridged Cyclic Lipopeptides. Org. Lett. 18 (16), 4096–4099. 10.1021/acs.orglett.6b02001 27505031

[B88] Münter R. Bak M. Christensen E. Kempen P. J. Larsen J. B. Kristensen K. (2022). Mechanisms of Selective Monocyte Targeting by Liposomes Functionalized with a Cationic, Arginine-Rich Lipopeptide. Acta Biomater. 10.1016/J.ACTBIO.2022.03.029 35314364

[B89] Murciano J. C. Higazi A. A. R. Cines D. B. Muzykantov V. R. (2009). Soluble Urokinase Receptor Conjugated to Carrier Red Blood Cells Binds Latent Pro-urokinase and Alters its Functional Profile. J. Control Release 139, 190–196. 10.1016/j.jconrel.2009.07.003 19616049 PMC2773161

[B90] Muthu M. S. Kutty R. Luo Z. Xie J. Feng S. S. (2015). Theranostic Vitamin E TPGS Micelles of Transferrin Conjugation for Targeted Co-delivery of Docetaxel and Ultra Bright Gold Nanoclusters. Biomaterials 39, 234–248. 10.1016/j.biomaterials.2014.11.008 25468374

[B91] Nanna A. R. Li X. Walseng E. Pedzisa L. Goydel R. S. Hymel D. (2017). Harnessing a Catalytic Lysine Residue for the One-step Preparation of Homogeneous Antibody-Drug Conjugates. Nat. Commun. 8 (1), 1112. 10.1038/s41467-017-01257-1 29062027 PMC5653646

[B92] NCT02195180 (2014). Efficacy and Safety of L-Asparaginase Encapsulated in RBC Combined with Gemcitabine or FOLFOX in 2nd Line for Progressive Metastatic Pancreatic Carcinoma. Available at: https://clinicaltrials.gov/show/nct02195180 .

[B93] NCT03267030 (2021) Asparaginase Encapsulated in Erythrocytes for Patients with ALL and Hypersensitivity to PEG-Asparaginase - Full Text View - ClinicalTrials.Gov. Available at: https://clinicaltrials.gov/ct2/show/NCT03267030 .

[B94] Nikezić A. V. V. Bondžić A. M. Vasić V. M. (2020). Drug Delivery Systems Based on Nanoparticles and Related Nanostructures. Eur. J. Pharm. Sci. 151, 105412. 10.1016/j.ejps.2020.105412 32505796

[B95] Ning Y.-M. He K. Dagher R. Sridhara R. Farrell A. T. Justice R. (2007). Liposomal Doxorubicin in Combination with Bortezomib for Relapsed or Refractory Multiple Myeloma. Oncology (Williston Park) 21, 1503–1508. discussion 1511, 1513, 1516 passim. 18077994

[B96] Ojediran J. O. Raji A. O. (2010). Chapter 2 Flow and Functional Models for Rheological Properties of Fluid Foods. Int. Food Res. J. 17, 1095–1106. 10.1007/978-1-4614-9230-6

[B97] Palko-Łabuz A. Gliszczyńska A. Skonieczna M. Poła A. Wesołowska O. Środa-Pomianek K. (2021). Conjugation with Phospholipids as a Modification Increasing Anticancer Activity of Phenolic Acids in Metastatic Melanoma—In Vitro and In Silico Studies. Int. J. Mol. Sci. 22, 8397. 10.3390/ijms22168397 34445104 PMC8395125

[B98] Pando O. Stark S. Denkert A. Porzel A. Preusentanz R. Wessjohann L. A. (2011). The Multiple Multicomponent Approach to Natural Product Mimics: Tubugis, N-Substituted Anticancer Peptides with Picomolar Activity. J. Am. Chem. Soc. 133 (20), 7692–7695. 10.1021/ja2022027 21528905

[B99] Papadopoulos G. N. Kokotos C. G. (2016). One-Pot Amide Bond Formation from Aldehydes and Amines via a Photoorganocatalytic Activation of Aldehydes. J. Org. Chem. 81, 7023–7028. 10.1021/acs.joc.6b00488 27227271

[B100] Parslow A. C. Parakh S. Lee F. T. Gan H. K. Scott A. M. (2016). Antibody-drug Conjugates for Cancer Therapy. Biomedicines 4, 17. 10.3390/biomedicines4030014 PMC5344263 28536381

[B101] Pérez-Labrada K. Brouard I. Méndez I. Rivera D. G. (2012). Multicomponent Synthesis of Ugi-type Ceramide Analogues and Neoglycolipids from Lipidic Isocyanides. J. Org. Chem. 77 (10), 4660–4670. 10.1021/jo300462m 22533639

[B102] Prasher P. Sharma M. Mudila H. Gupta G. Sharma A. K. Kumar D. (2020). Emerging Trends in Clinical Implications of Bio-Conjugated Silver Nanoparticles in Drug Delivery. Colloids Interf. Sci. Commun. 35, 100244. 10.1016/j.colcom.2020.100244

[B103] Ramos Tomillero I. (2016). Linkers for Bioconjugation PhD thesis. Available at: http://hdl.handle.net/10803/399915 (Accessed May 2, 2022).

[B104] Rivera D. G. León F. Concepción O. Morales F. E. Wessjohann L. A. (2013). A Multiple Multicomponent Approach to Chimeric Peptide-Peptoid Podands. Chem. - A. Eur. J. 19 (20), 6417–6428. 10.1002/chem.201201591 23512744

[B105] Rivera D. G. Pando O. Coll F. (2006). Synthesis of Peptidomimetic-Spirostane Hybrids via Ugi Reaction: a Versatile Approach for the Formation of Peptide-Steroid Conjugates. Tetrahedron 62 (35), 8327–8334. 10.1016/j.tet.2006.06.050

[B106] Rivera D. G. Pérez-Labrada K. Lambert L. Dörner S. Westermann B. Wessjohann L. A. (2012). Carbohydrate-steroid Conjugation by Ugi Reaction: One-Pot Synthesis of Triple Sugar/pseudo-Peptide/spirostane Hybrids. Carbohydr. Res. 359, 102–110. 10.1016/j.carres.2012.05.003 22925772

[B107] Rossi L. Pierigè F. Aliano M. P. Magnani M. (2020). Ongoing Developments and Clinical Progress in Drug-Loaded Red Blood Cell Technologies. BioDrugs 34, 265–272. 10.1007/s40259-020-00415-0 32198632 PMC7211199

[B108] Samavarchi-Tehrani P. Samson R. Gingras A.-C. (2020). Proximity Dependent Biotinylation: Key Enzymes and Adaptation to Proteomics Approaches. Mol. Cel. Proteomics 19, 757–773. 10.1074/MCP.R120.001941 PMC7196579 32127388

[B109] Saw W. S. Anasamy T. Foo Y. Y. Kwa Y. C. Kue C. S. Yeong C. H. (2021). Delivery of Nanoconstructs in Cancer Therapy: Challenges and Therapeutic Opportunities. Adv. Ther. 4, 2000206. 10.1002/adtp.202000206

[B110] Sen S. Perrin M. W. Sedgwick A. C. Lynch V. M. Sessler J. L. Arambula J. F. (2021). Covalent and Non-covalent Albumin Binding of Au(i) Bis-NHCsviapost-Synthetic Amide Modification. Chem. Sci. 12, 7547–7553. 10.1039/d1sc01055g 34163845 PMC8171490

[B111] Sharma S. Kaur G. Handa S. (2021). Insights into Fast Amide Couplings in Aqueous Nanomicelles. Org. Process. Res. Dev. 25, 1960–1965. 10.1021/acs.oprd.1c00203

[B112] Singla P. Salunke D. B. (2020). Recent Advances in Steroid Amino Acid Conjugates: Old Scaffolds with New Dimensions. Eur. J. Med. Chem. 187, 111909. 10.1016/j.ejmech.2019.111909 31830636

[B113] Sleep D. (2015). Albumin and its Application in Drug Delivery. Expert Opin. Drug Deliv. 12, 793–812. 10.1517/17425247.2015.993313 25518870

[B114] Sonali Singh R. P. S. Singh N. Sharma G. Vijayakumar M. R. Koch B. (2016). Transferrin Liposomes of Docetaxel for Brain-Targeted Cancer Applications: Formulation and Brain Theranostics. Drug Deliv. 23, 1261–1271. 10.3109/10717544.2016.1162878 26961144

[B115] Spanedda M. V. Bourel-Bonnet L. (2021). Cyclic Anhydrides as Powerful Tools for Bioconjugation and Smart Delivery. Bioconjug. Chem. 32, 482–496. 10.1021/acs.bioconjchem.1c00023 33662203

[B117] Taguchi K. Lu H. Jiang Y. Hung T. T. Stenzel M. H. (2018). Safety of Nanoparticles Based on Albumin-Polymer Conjugates as a Carrier of Nucleotides for Pancreatic Cancer Therapy. J. Mater. Chem. B 6, 6278–6287. 10.1039/C8TB01613E 32254618

[B118] Tang L. Li J. Zhao Q. Pan T. Zhong H. Wang W. (2021). Advanced and Innovative Nano-Systems for Anticancer Targeted Drug Delivery. Pharmaceutics 13, 1151. 10.3390/pharmaceutics13081151 34452113 PMC8398618

[B119] Tanino T. Ichikawa S. Al-Dabbagh B. Bouhss A. Oyama H. Matsuda A. (2010). Synthesis and Biological Evaluation of Muraymycin Analogues Active against Anti-drug-resistant Bacteria. ACS Med. Chem. Lett. 10.1021/ml100057z PMC4007965 24900205

[B120] Thery J. C. Spano J. P. Azria D. Raymond E. Penault Llorca F. (2014). Resistance to Human Epidermal Growth Factor Receptor Type 2-targeted Therapies. Eur. J. Cancer 50, 892–901. 10.1016/j.ejca.2014.01.003 24462377

[B121] Tivdak Fda. Initial U.S. Approval: 2021 (2021). TIVDAK TM (Tisotumab Vedotin-Tftv) for Injection, for Intravenous Use Initial U.S. Approval: 2021. Ref. ID 4859741. Available at: https://www.accessdata.fda.gov/drugsatfda_docs/label/2021/761208s000lbl.pdf (Accessed February 25, 2022).

[B122] Tolaney S. M. Do K. T. Eder J. P. LoRusso P. M. Weekes C. D. Chandarlapaty S. (2021). A Phase I Study of DLYE5953A, an Anti-ly6e Antibody Covalently Linked to Monomethyl Auristatin E, in Patients with Refractory Solid Tumors. Clin. Cancer Res. 26, 5588–5597. 10.1158/1078-0432.CCR-20-1067 PMC9899652 32694157

[B123] Tong J. T. W. Harris P. W. R. Brimble M. A. Kavianinia I. (2021). ZYNLONTA^TM^ (Loncastuximab Tesirine-Lpyl) for Injection, for Intravenous Use Initial U.S. Approval: 2021. Mol 26, 5847. *Page 5847* 26. 10.3390/MOLECULES26195847

[B124] Torres-Pérez S. A. Torres-Pérez C. E. Pedraza-Escalona M. Pérez-Tapia S. M. Ramón-Gallegos E. (2020). Glycosylated Nanoparticles for Cancer-Targeted Drug Delivery. Front. Oncol. 10, 2667. 10.3389/FONC.2020.605037/BIBTEX PMC7735155 33330106

[B125] Tsuchikama K. An Z. (2018). Antibody-drug Conjugates: Recent Advances in Conjugation and Linker Chemistries. Protein Cell 9, 33–46. 10.1007/s13238-016-0323-0 27743348 PMC5777969

[B126] Tsukigawa K. Imoto S. Yamasaki K. Nishi K. Tsutsumi T. Yokoyama S. (2020). Synthesis and *In Vitro* Assessment of pH-Sensitive Human Serum Albumin Conjugates of Pirarubicin. Pharm 202114, 22. *Page 22* 14. 10.3390/PH14010022 PMC7823624 33396604

[B127] United States Environmental Protection Agency (2021). Research on Nanomaterials | US EPA. US EPA. Available at: https://www.epa.gov/chemical-research/research-nanomaterials (Accessed May 2, 2022).

[B128] U.S. Food and Drug Administration (FDA) (2013). FDA Approves Kadcyla for Breast Cancer. Cancer Discov. 3, 366–2366. 10.1158/2159-8290.cd-nb2013-030

[B129] U.S. Food and Drug Administration (FDA) (2020). Novel Drug Approvals for 2020. U.S. Food Drug Adm. Available at: https://www.fda.gov/drugs/new-drugs-fda-cders-new-molecular-entities-and-new-therapeutic-biological-products/novel-drug-approvals-2020.

[B130] U.S. Food and Drug Admins (1995). DOXIL® (doxorubicin HCl liposome injection) for intravenous infusion. Available at: https://www.fda.gov/medwat (Accessed May 2, 2022).

[B131] U.S. Food and Drug Admins (2010). KRYSTEXXATM® (Pegloticase) Injection, for Intravenous Infusion. Available at: https://www.fda.gov/media/79472/download (Accessed May 2, 2022).

[B132] U.S. Food and Drug Admins (1994). ONCASPAR (Pegaspargase) Injection, for Intramuscular or Intravenous Use. Available at: https://www.accessdata.fda.gov/drugsatfda_docs/label/2021/103411s5201lbl.pdf (Accessed May 2, 2022).

[B133] U.S. Food and Drug Admins (2015). PLEGRIDY (Peginterferon Beta-1a) Injection, for Subcutaneous Injection. Available at: https://www.accessdata.fda.gov/drugsatfda_docs/label/2014/125499lbl.pdf (Accessed May 2, 2022).

[B134] U.S. Food and Drug Admins (2012a). Temporary Importation of Lipodox (Doxorubicin HCL Liposomal Injection). Available at: http://www.fda.gov/downloads/Drugs/DrugSafety/DrugShortages/UCM292634.pdf (Accessed May 2, 2022).

[B135] U.S. Food and Drug Adminstration (FDA) (2012b). ADCETRIS® (Brentuximab Vedotin) for injection, for intravenous use. Available at: https://www.accessdata.fda.gov/drugsatfda_docs/label/2014/125388_s056s078lbl.pdf (Accessed May 2, 2022).

[B136] U.S. Food and Drug Adminstration (FDA) (2017a). BESPONSA (Inotuzumab Ozogamicin). Available at: https://www.accessdata.fda.gov/drugsatfda_docs/label/2017/761040s000lbl.pdf (Accessed May 2, 2022).

[B137] U.S. Food and Drug Adminstration (FDA) (2017b). MYLOTARGT (Gemtuzumab Ozogamicin) for Injection. Available at: https://www.accessdata.fda.gov/drugsatfda_docs/label/2017/761060lbl.pdf (Accessed May 2, 2022).

[B138] Vega-Vásquez P. Mosier N. S. Irudayaraj J. (2020). Nanoscale Drug Delivery Systems: From Medicine to Agriculture. Front. Bioeng. Biotechnol. 8, 79. 10.3389/FBIOE.2020.00079/BIBTEX 32133353 PMC7041307

[B21] Villa C. H. Cines D. B Siegel D. L. Muzykantov V. (2017). Erythrocytes as Carriers for Drug Delivery in Blood Transfusion and beyond. Transfus. Med. Rev. 31, 26–35. 10.1016/J.TMRV.2016.08.004 27707522 PMC5161683

[B139] Volpi S. Cancelli U. Neri M. Corradini R. (2021). Multifunctional Delivery Systems for Peptide Nucleic Acids. Pharmaceuticals 14, 1–31. 10.3390/ph14010014 PMC7823687 33375595

[B140] Walther R. Zelikin A. N. (2021). Chemical (Neo)glycosylation of Biological Drugs. Adv. Drug Deliv. Rev. 171, 62–76. 10.1016/J.ADDR.2021.01.021 33548302

[B141] Wang C. Sun X. Cheng L. Yin S. Yang G. Li Y. (2014). Multifunctional Theranostic Red Blood Cells for Magnetic-Field-Enhanced *In Vivo* Combination Therapy of Cancer. Adv. Mater. 26, 4794–4802. 10.1002/ADMA.201400158 24838472

[B142] Wang K. Shang F. Chen D. Cao T. Wang X. Jiao J. (2021). Protein Liposomes-Mediated Targeted Acetylcholinesterase Gene Delivery for Effective Liver Cancer Therapy. J. Nanobiotechnology 19, 1–15. 10.1186/s12951-021-00777-9 33482834 PMC7821407

[B81] Weisel J. W. Litvinov R. I. (2019). Red Blood Cells: The Forgotten Player in Hemostasis and Thrombosis J. Thromb. Haemost. 17, 271–282. 10.1111/JTH.14360 30618125 PMC6932746

[B143] Werengowska-Ciećwierz K. Wis̈niewski M. Terzyk A. P. Furmaniak S. (2015). The Chemistry of Bioconjugation in Nanoparticles-Based Drug Delivery System. Adv. Condens. Matter Phys. 2015, 1–27. 10.1155/2015/198175

[B144] Wessjohann L. A. Morejón M. C. Ojeda G. M. Rhoden C. R. B. Rivera D. G. (2016). Applications of Convertible Isonitriles in the Ligation and Macrocyclization of Multicomponent Reaction-Derived Peptides and Depsipeptides. J. Org. Chem. 10.1021/acs.joc.6b01150 27390908

[B145] Whalley N. A. Walters S. Hammond K. (2018). “Molecular Cell Biology,” in Molecular Medicine for Clinicians (W. H. & C. Freeman), 37–49. 10.18772/22008014655.9

[B146] World Health Organization (2018). WHO | Key Facts about Cancer. Geneva, Switzerland: WHO.

[B147] Wu B. Melhem M. Subramanian R. Chen P. Jaramilla Sloey B. Fouqueray B. (2018). Clinical Pharmacokinetics and Pharmacodynamics of Etelcalcetide, a Novel Calcimimetic for Treatment of Secondary Hyperparathyroidism in Patients with Chronic Kidney Disease on Hemodialysis. J. Clin. Pharmacol. 58, 717–726. 10.1002/jcph.1090 29534286

[B148] Xu Y. D. Tian L. Lai R. Y. Li Z. Procházková E. Ho J. (2022). Development of an Albumin-Polymer Bioconjugate via Covalent Conjugation and Supramolecular Interactions. Bioconjug. Chem. 33, 321–332. 10.1021/ACS.BIOCONJCHEM.1C00536/SUPPL_FILE/BC1C00536_SI_001.PDF 35057618

[B149] Xue Y. Bai H. Peng B. Fang B. Baell J. Li L. (2021). Stimulus-cleavable Chemistry in the Field of Controlled Drug Delivery. Chem. Soc. Rev. 50, 4872–4931. 10.1039/D0CS01061H 33734247

[B150] Yamazaki C. M. Yamaguchi A. Anami Y. Xiong W. Otani Y. Lee J. (2021). Antibody-drug Conjugates with Dual Payloads for Combating Breast Tumor Heterogeneity and Drug Resistance. Nat. Commun. 12, 1–13. 10.1038/s41467-021-23793-7 34112795 PMC8192907

[B151] Yang W. Lee J. C. Chen M. H. Zhang Z. Y. Bai X. M. Yin S. S. (2019). Thermosensitive Liposomal Doxorubicin Plus Radiofrequency Ablation Increased Tumor Destruction and Improved Survival in Patients with Medium and Large Hepatocellular Carcinoma: A Randomized, Double-Blinded, Dummy-Controlled Clinical Trial in a Single Cent. J. Cancer Res. Ther. 15, 773–783. 10.4103/jcrt.JCRT_801_18 31436231

[B152] Yao W. Liu C. Wang N. Zhou H. Chen H. Qiao W. (2021). An MRI-Guided Targeting Dual-Responsive Drug Delivery System for Liver Cancer Therapy. J. Colloid Interf. Sci. 603, 783–798. 10.1016/j.jcis.2021.06.151 34246838

[B153] Zakharov Y. N. Raevski I. P. Eknadiosians E. I. Pinskaya A. N. Pustovaya L. E. Borodin V. Z. (2000). Pyroelectric Properties and Domain Structure of Modified lead Ferroniobate-Based Ceramics. Ferroelectrics 247, 47–52. 10.1080/00150190008214939

[B154] Zhang L. Wang Z. Wang Z. Luo F. Guan M. Xu M. (2021a). A Simple and Efficient Method to Generate Dual Site-specific Conjugation ADCs with Cysteine Residue and an Unnatural Amino Acid. Bioconjug. Chem. 32, 1094–1104. 10.1021/acs.bioconjchem.1c00134 34013721

[B155] Zhang X. Luo M. Dastagir S. R. Nixon M. Khamhoung A. Schmidt A. (2021b). Engineered Red Blood Cells as an Off-The-Shelf Allogeneic Anti-tumor Therapeutic. Nat. Commun. 12, 1–14. 10.1038/s41467-021-22898-3 33976146 PMC8113241

[B156] Zhang Y.-T. He K.-J. Zhang J.-B. Ma Q.-H. Wang F. Liu C.-F. (2021c2021). Advances in Intranasal Application of Stem Cells in the Treatment of central Nervous System Diseases. Stem Cel Res. Ther. 121 12, 1–10. 10.1186/S13287-021-02274-0 PMC7992869 33762014

[B157] Zhang Y. Xia Q. Wu T. He Z. Li Y. Li Z. (2021d). A Novel Multi-Functionalized Multicellular Nanodelivery System for Non-small Cell Lung Cancer Photochemotherapy. J. Nanobiotechnology 19, 1–23. 10.1186/s12951-021-00977-3 34391438 PMC8364713

[B158] Ziegler T. Gerling S. Lang M. (2000). Preparation of Bioconjugates through an Ugi Reaction. Angew. Chem. - Int. Ed. 10.1002/1521-3773(20000616)39:12<2109::aid-anie2109>3.0.co;2-9 10941031

[B159] Zielińska A. Szalata M. Gorczyński A. Karczewski J. Eder P. Severino P. (2021). Cancer Nanopharmaceuticals: Physicochemical Characterization and *In Vitro*/*In Vivo* Applications. Cancers (Basel) 13, 1896. 10.3390/cancers13081896 33920840 PMC8071188

[B160] Znabet A. Polak M. M. Janssen E. De Kanter F. J. J. Turner N. J. Orru R. V. A. (2010). A Highly Efficient Synthesis of Telaprevir by Strategic Use of Biocatalysis and Multicomponent Reactions. Chem. Commun. 46 (42), 7918. 10.1039/c0cc02823a 20856952

[B161] Zynlonta Fda. Initial U.S. Approval: 2021 (2021). ZYNLONTA^TM^ (Loncastuximab Tesirine-Lpyl) for Injection, for Intravenous Use. *Ref. ID 4784313* . Available at: https://www.accessdata.fda.gov/drugsatfda_docs/label/2021/761196s000lbl.pdf (Accessed February 25, 2022).

